# Identification of Putative Transmembrane Proteins Involved in Salinity Tolerance in *Chenopodium quinoa* by Integrating Physiological Data, RNAseq, and SNP Analyses

**DOI:** 10.3389/fpls.2017.01023

**Published:** 2017-06-21

**Authors:** Sandra M. Schmöckel, Damien J. Lightfoot, Rozaimi Razali, Mark Tester, David E. Jarvis

**Affiliations:** ^1^Division of Biological and Environmental Sciences and Engineering, King Abdullah University of Science and TechnologyThuwal, Saudi Arabia; ^2^Computational Bioscience Research Center, King Abdullah University of Science and TechnologyThuwal, Saudi Arabia

**Keywords:** quinoa, *Chenopodium quinoa*, hydroponics, salinity tolerance, RNAseq, plant physiology, transporters, comparative genomics

## Abstract

*Chenopodium quinoa* (quinoa) is an emerging crop that produces nutritious grains with the potential to contribute to global food security. Quinoa can also grow on marginal lands, such as soils affected by high salinity. To identify candidate salt tolerance genes in the recently sequenced quinoa genome, we used a multifaceted approach integrating RNAseq analyses with comparative genomics and topology prediction. We identified 219 candidate genes by selecting those that were differentially expressed in response to salinity, were specific to or overrepresented in quinoa relative to other Amaranthaceae species, and had more than one predicted transmembrane domain. To determine whether these genes might underlie variation in salinity tolerance in quinoa and its close relatives, we compared the response to salinity stress in a panel of 21 *Chenopodium* accessions (14 *C. quinoa*, 5 *C. berlandieri*, and 2 *C. hircinum*). We found large variation in salinity tolerance, with one *C. hircinum* displaying the highest salinity tolerance. Using genome re-sequencing data from these accessions, we investigated single nucleotide polymorphisms and copy number variation (CNV) in the 219 candidate genes in accessions of contrasting salinity tolerance, and identified 15 genes that could contribute to the differences in salinity tolerance of these *Chenopodium* accessions.

## Introduction

Soil salinization is a major threat to agriculture, affecting approximately 20% of irrigated land and causing a substantial reduction in crop yield (Qadir et al., 2014). The impact of reduced yields is aggravated by a continuously increasing human population and rising food security concerns. To meet future world food demands, the current rate of yield increase must increase by 37% by 2050 (Tester and Langridge, 2010). The increased use of marginal soils, such as salt affected lands that are currently not fully utilized, has the potential to contribute to increased yield. *Chenopodium quinoa* (quinoa) is a favorable candidate for agronomic expansion into these marginal lands and for identification of candidate genes facilitating salinity tolerance because it is naturally adapted to marginal environments (such as the high saline plains in the Andean Altiplano in Bolivia and Peru and the coastal regions of Chile), it is relatively salt tolerant and it produces highly nutritious grains (Hariadi et al., 2011; Gordillo-Bastidas et al., 2016). The potential of this emerging crop was recognized by the United Nations when 2013 was declared the International Year of Quinoa.

Quinoa belongs to the Amaranthaceae, which also includes *Beta vulgaris* (beet), *Amaranthus hypochondriacus* (amaranth), and *Spinacia oleracea* (spinach). Within the Amaranthaceae, there is large variation in salinity tolerance between species. For instance, beet and quinoa have high salinity tolerances whereby their relative growth rate is reduced by approximately 30% at 300 mM NaCl (Rozema et al., 2015), whereas amaranth and spinach have lower tolerances to salt stress, showing similar growth reductions (based on fresh mass) at only 170 and 120 mM NaCl, respectively (Qin et al., 2013; Ors and Suarez, 2016). While quinoa has been characterized as being salt tolerant (Rozema et al., 2015), little is known about the molecular and physiological basis of this tolerance. The recent publication of the quinoa genome (Jarvis et al., 2017) now enables a genome-wide investigation of the high salt tolerance of the species.

Plants have evolved three major mechanisms to tolerate salt stress. The first mechanism is osmotic tolerance, which is the ability to maintain growth during the initial stages of salinity stress through mechanisms which are as yet, primarily, unknown (Munns and Tester, 2008; Roy et al., 2014). The second and third mechanisms, Na^+^ exclusion and Na^+^ tissue tolerance, facilitate reduction of the concentration of Na^+^ in the cytosol, especially in photosynthetically active leaves. It has been shown that Na^+^ exclusion can be mediated by transporters such as SOS1 and HKT1;1, which are involved in reducing the amount of Na^+^ being translocated from the root to the shoot, thereby excluding it from the photosynthetically active tissues (Apse and Blumwald, 2007; Deinlein et al., 2014). AtHKT1;1, for instance, is proposed to be localized in the root stele, with a role in retention of Na^+^ in the root, thus preventing Na^+^ from reaching the shoot *via* the transpiration stream (Møller and Tester, 2007). Tissue tolerance refers to the mechanism of compartmentalizing Na^+^ into vacuoles, thereby removing it from the cytoplasm. It is proposed that sodium/proton (Na^+^/H^+^) antiporters are involved in the transport of Na^+^ across the tonoplast (Apse et al., 1999; Pardo et al., 2006). While the Na^+^/H^+^ antiporter activity was initially attributed to NHX1 (Apse et al., 2003), recent research indicates that NHX1 may be a K^+^/H^+^ antiporter (Jiang et al., 2010; Leidi et al., 2010; Bassil et al., 2011; Barragán et al., 2012).

To identify novel genes that might contribute to salinity tolerance in quinoa and/or the *Chenopodium* genus, we integrated several complementary bioinformatics approaches with physiological measurements of plant growth under salt stress. We used RNAseq to identify genes that are differentially expressed in response to salt stress in the reference genome accession, PI 614886 (Jarvis et al., 2017). In parallel, we focused on genes that are unique to or overrepresented in quinoa (and those that are also overrepresented in beet) relative to other members of the Amaranthaceae, as these genes may underlie the high salinity tolerance of quinoa. Additionally, because transporters have previously been shown to play crucial roles in salinity tolerance (Roy et al., 2014; Volkov, 2015), we focused on genes that are predicted to encode transmembrane proteins (which include transporters) by selecting genes with more than one predicted transmembrane domain (TMD). By integrating these approaches, we identified a total of 219 preliminary candidate genes that are responsive to salinity, unique to or overrepresented in quinoa (and beet) and that contain more than one putative TMD. We then investigated whether these genes might contribute to variation in salinity tolerance in quinoa and its close relatives by characterizing the response of 21 *Chenopodium* accessions [14 accessions of quinoa (8 highland and 6 coastal), 5 accessions of *C. berlandieri* and 2 accessions of *C. hircinum*] to salt stress and observed phenotypic variation in salinity tolerance between quinoa accessions, as has been previously reported (Hariadi et al., 2011; Morales et al., 2011; Ruiz-Carrasco et al., 2011; Adolf et al., 2012; Ruiz et al., 2016). We utilized this variation in salinity tolerance to investigate copy number variation (CNV) and single-nucleotide polymorphisms (SNPs) in the 219 preliminary candidate genes in the accessions that showed the highest and the lowest levels of salinity tolerance.

While several previous studies have utilized a subset of these approaches (Czaban et al., 2015; Frades et al., 2015; Guo et al., 2015; Tsukagoshi et al., 2015), here we present an extension of those methodologies to more fully integrate comparative genomics, gene expression data, and predicted protein topology with extensive physiological characterizations. This approach resulted in the identification of 15 candidate genes with CNV or SNP variants that might be associated with the tolerant *Chenopodium* accessions. These 15 candidate genes might contribute to the variation in salinity tolerance of quinoa and represent promising candidates for further investigation.

## Materials and methods

### RNAseq

RNA was extracted from the reference genome quinoa accession PI 614886 grown hydroponically under control conditions or exposed to 300 mM NaCl for 7 days. The hydroponic growth system was based on Conn et al. (2013). Briefly, seeds were sown on germination medium containing 0.7% agar and grown for 2 weeks in tanks containing basal nutrient solution (BNS) exactly as described by Conn et al. (2013). Plants were then transferred to larger aerated tanks containing BNS. After one additional week of growth, plants were either transferred to tanks containing fresh BNS (control) or tanks containing fresh BNS supplemented with 150 mM NaCl (salinity). After 24 h, the NaCl concentration in the salinity treatment was increased to 300 mM. One week after the start of the treatment, all below-ground tissues (defined here as roots) and all above-ground tissues (defined here as shoots) were harvested separately and snap frozen in liquid nitrogen. RNA was isolated from these tissues using the Zymo Direct-zol RNA MiniPrep Kit. RNA quality was assessed using an Agilent 2100 BioAnalyzer. Sequencing libraries were prepared using the NEBNext Ultra Directional RNA Library Prep Kit for Illumina. Paired-end sequencing of 100-bp was performed using an Illumina HiSeq 2000 at KAUST. Three biological replicates for each treatment and each tissue were sequenced, but one sample for salt treated roots was excluded as it did not pass quality control.

An average of 10.8 million paired reads were generated for each sample. Sequencing reads were processed with Trimmomatic (v0.33) (Bolger et al., 2014) to remove adapter sequences, leading and trailing bases with a quality score below 20 and reads with an average per base quality of 20 over a 5-bp sliding window. Reads less than 50 nucleotides in length after trimming were removed from further analysis, and the remaining high-quality reads were mapped to the quinoa reference genome assembly (Jarvis et al., 2017) using TopHat (Trapnell et al., 2009). Genes differentially expressed between shoots of control and salt-treated plants and between roots of control and salt-treated plants were identified using default parameters of the Cuffdiff function of the Cufflinks program (Trapnell et al., 2010).

Gene ontology (GO) terms were previously assigned to some of the annotated genes in the quinoa genome (Jarvis et al., 2017). Of the genes differentially expressed between salt and control treatments, GO terms were assigned to 2,984 and 975 differentially expressed in the shoot and root, respectively. Among these, 2,975 GO terms were classified into molecular function, 693 into biological process and 291 into cellular component. Analysis of enriched GO terms in the differentially expressed genes was performed using FunRich (Pathan et al., 2015). Fold changes were calculated using a background database of GO terms from all annotated quinoa genes expressed in shoots or roots of control and salt-treated plants.

### Identification of quinoa overrepresented genes

Orthologous and paralogous gene clusters were identified as described in Jarvis et al. (2017) using OrthoMCL (Li et al., 2003) with the following species: *Chenopodium quinoa, Beta vulgaris, Spinacia oleracea*, and *Amaranthus hypochondriacus*. From the OrthoMCL analysis, we selected two different classes of genes for further analysis; (1) genes specific to quinoa with no identified homologs (orthologs or paralogs) in the four Amaranthaceae genomes; (2) clusters of genes that are overrepresented in quinoa (and beet). This second class of candidates contains two subclasses. Firstly, clusters of genes that have paralogs in quinoa but no orthologs in the other 3 genomes. The second subclass contains gene clusters that show an increased abundance of quinoa (and beet) genes in the cluster relative to the other species. Because quinoa and amaranth are tetraploids, two copies (homoeologs) of each gene may be present in the genome relative to the diploid species, beet and spinach. Therefore, gene clusters with an overrepresentation of quinoa genes were defined as clusters with more quinoa genes than amaranth genes and also with more than double the number of quinoa genes relative to the number of genes from both spinach and beet. Gene clusters with an overrepresentation of quinoa and beet genes were defined as clusters that had more quinoa genes than amaranth genes and more than double the number of quinoa genes relative to spinach genes as well as more beet genes than spinach genes and more than double the number of beet genes relative to quinoa. Clusters with an overrepresentation of both quinoa and beet genes were selected for further analysis as beet is known to possess a high salinity tolerance and, as such, it is possible that gene families that encode genes related to salinity tolerance may have undergone expansion in both quinoa and beet. The composition of the gene family clusters was analyzed using custom Perl scripts and the ratio of genes from each species within the gene family clusters was plotted using JMP. Where the 2-way ratio between the numbers of genes in two different species within one gene family had an infinite value, for the purpose of visualization, the data point was assigned the value corresponding to the maximum ratio found within the corresponding 2-way comparison.

### Prediction of transmembrane domains

The number of TMDs for all annotated quinoa genes was predicted using TMHMM version 2.0 (Krogh et al., 2001) *via* the Center for Biological Sequence Analysis server (http://www.cbs.dtu.dk/services/TMHMM/). Custom scripts were used to select proteins with more than one predicted TMD.

### Plant phenotyping

**Figure 1:**
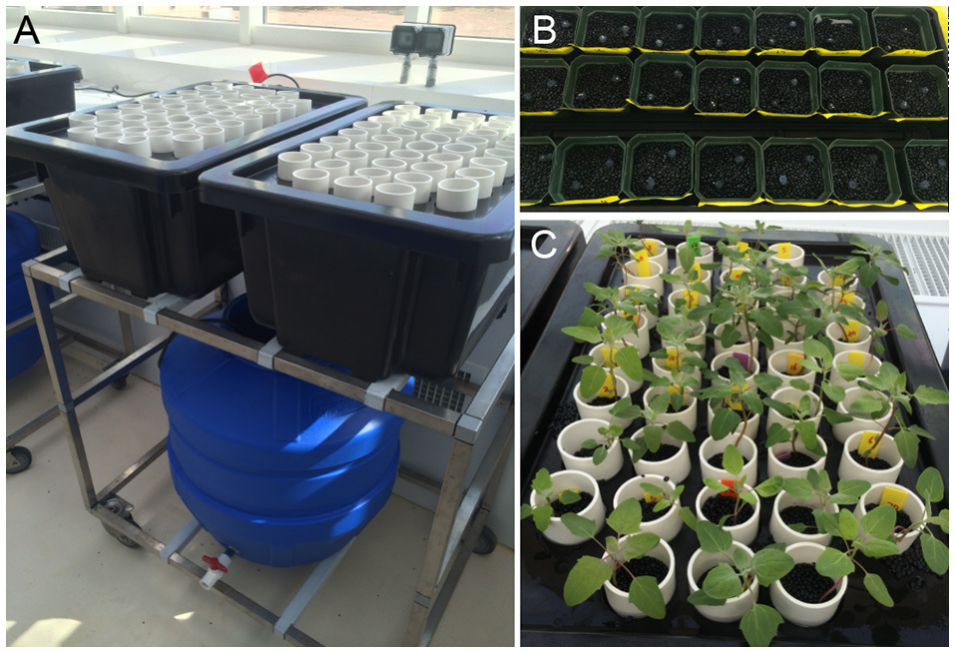
Hydroponics system setup. **(A)** Trolley holding the blue reservoir tank (containing 100 L of nutrient solution) is located below two black plant tanks, each holding 42 white tubes filled with round black plastic beads to create a matrix holding the plant. A pump enables flooding and draining of the plant tanks in 20-min intervals. **(B)** Agar plugs carrying the seeds were inserted into the matrix and covered with nutrient solution for germination. **(C)** Plants growing in the matrix. Photo was taken on the day of salt imposition.

In total, 21 accessions described in Jarvis et al. (2017) were assessed for traits of salinity tolerance (Table 1; for simplicity, the numbering system in Table 1 is used throughout this document). Plants were grown hydroponically in a supported system described for barley by Shavrukov et al. (2012). Plants were grown in the greenhouse at 24°C/22°C day/night with a day length of approximately 11.5 h. Plants were germinated on agar plugs (½ Murashige and Skoog medium, 1% phyto-agar, pH 5.8) resting in plastic beads covered with nutrient solution (0.2 mM NH_4_NO_3_, 5 mM KNO_3_, 2 mM Ca(NO_3_)_2_, 2 mM MgSO_4_, 0.1 mM KH_2_PO_4_, 0.1 mM NaFe(III)EDTA, 25 μM KCl, 12 μM H_3_BO_3_, 2 μM MnCl_2_, 3 μM ZnSO_4_, 0.5 μM CuSO_4_, 0.1 μM Na_2_MoO_4_, 0.1 μM NiSO_4_, pH 5.8). When seedlings were at the 4–5 leaf stage (approximately 18 days for most accessions), plants were transferred to the supported hydroponics system filled with the nutrient solution (Figure 1). Ebb and flow was set to 20 min, which allowed the system to fully drain and aerate roots before it was refilled. The pH was monitored throughout the experiment and remained at 5.7–5.9.

**Table 1:** *Chenopodium* accessions used to investigate salinity tolerance.

	**Species**	**Accession**	**Origin**	**Type**	**Ecotype**
1	*C. q*.	0654	Peru	Highland	Altiplano
2	*C. b*. subsp. *nuttalliae*	PI 568156	Mexico		Cultivated (huauzontle)
3	*C. b*. var. *boscianum*	BYU 937	Texas, US		Wild/weedy
4	*C. b*. var. *macrocalycium*	PI 666279	Maine, US		Wild/weedy
5	*C. b*. var. *sinuatum*	Ames 33013	Arizona, US		Wild/weedy
6	*C. b*. var. *zschackei*	BYU 1314	Utah, US		Wild/weedy
7	*C. h*.	BYU 1101	Argentina		Weedy (pampas)
8	*C. h*.	BYU 566	Chile		Weedy (desert valley)
9	*C. q*.	CICA-17	Peru	Highland	Andean Valley
10	*C. q*.	G-205-95DK	Denmark	Coastal	Coastal
11	*C. q*.	Ollague	Chile	Highland	Salares
12	*C. q*.	Pasankalla	Peru	Highland	Andean Valley
13	*C. q*.	Real	Bolivia	Highland	Salares
14	*C. q*.	Regalona	Chile	Coastal	Coastal
15	*C. q*.	Salcedo INIA	Peru	Highland	Altiplano
16	*C. q*.	Cherry Vanilla	Oregon, US	Coastal	Coastal
17	*C. q*.	Ku-2	Chile	Coastal	Coastal
18	*C. q*.	Chucapaca	Bolivia	Highland	Altiplano
19	*C. q*.	PI 634921	Chile	Coastal	Coastal
20	*C. q*.	Kurmi	Bolivia	Highland	Altiplano
21	*C. q*.	PI 614886	Chile	Coastal	Coastal

*C.q., Chenopodium quinoa; C.b., Chenopodium berlandieri; C.h., Chenopodium hircinum*.

Assessment of salinity tolerance and analyses were performed as recommended by Negrão et al. (2016). To ensure plants were at a comparable developmental stage at the time salinity stress was imposed, the four accessions with delayed germination, *C. berlandieri* var. *boscianum, C. berlandieri* var. *macrocalycium, C. hircinum* BYU 566, and *C. hircinum* BYU 1101, were sown 6 days earlier than all other accessions.

To better evaluate the response to salinity, a destructive harvest of plants was performed before salinity imposition (t_0_). Fresh and dry root and shoot masses, root length and leaf area were determined in four biological replicates. Salt was applied 7 days after plants were transferred to the tanks. A total of 300 mM NaCl was applied in 50 mM increments every 12 h. Calcium (in the form of CaCl_2_) was added to the salt treated tanks to maintain the calcium activity, as calculated using GeochemEZ (Schaff et al., 2010). Leaf area measurements were taken using the scanning software WinFOLIA (Régent Instruments Inc.). Once plants were subjected to destructive harvest, roots and shoots were separated. Roots were rinsed twice in 20 mM MgSO_4_ for 2 s and dried on tissue paper for 3 s and then root length was determined using a ruler; roots were weighed and used for flame photometer analysis.

### Determination of sodium and potassium concentrations

Half of the root (divided longitudinally) and one leaf, which appeared to be the youngest fully expanded leaf (and therefore developed during salinity stress), were digested in 10 mL of 1% nitric acid at 60°C overnight. Na and K measurements were taken using a Model 420 flame photometer (Sherwood Scientific Ltd., Cambridge, UK).

Salt Tolerance (ST) index was calculated using the Equation (1):

(1) $$\begin{array}{r} ST=\frac{T_{salt}-T_{before\;treatment}}{T_{control}-T_{before\;treatment}}, \end{array}$$

where T is the observed trait (e.g., shoot biomass) before or after treatment.

All statistical analyses were performed in JMP. Statistical comparisons were made using log-transformed data and outliers were removed based on normal quantile plots. The data were fitted using standard least squares and restricted maximum likelihood estimations (REML). Fixed effects were chosen as Treatment (0 or 300 mM NaCl) and Line (21 accessions) and the interaction Treatment x Line. The trolley on which plants were grown was chosen as a random effect.

### Comparing copy number variation for candidate genes in quinoa accessions

After exposure to salt stress, we selected the four quinoa accessions with the highest and the four with the lowest fresh mass ST index for use in the CNV analysis. Analysis was performed using CNV-seq (Xie and Tammi, 2009) to generate 16 comparison results between the two sets of quinoa accessions to identify regions of genomic amplification or deletion using the following settings: *p* ≤ 0.001, log_2_ threshold ≥ 0.6, window size = 5, minimum windows required = 10 and genome-size 1,385,456,844 bp. Test and reference samples (short-read sequencing data of the selected quinoa accessions) were aligned to the quinoa template genome (accession PI 614868, Jarvis et al., 2017) using the Burrows–Wheeler aligner (ver 0.7.10) (Li and Durbin, 2009) and processing with SAMtools (ver 1.3.1) (Li et al., 2009). For each comparison, we used the more salt tolerant samples as the test set and the less salt tolerant samples as the reference set. CNVs in the 219 initially identified candidate genes were selected by filtering for hits with log_2_ ≥ 1 or ≤ −1. The average log_2_ was calculated for gene regions with multiple CNVs.

Because the efficiency of mapping sequencing reads from *C. berlandieri* and *C. hircinum* onto the quinoa reference genome assembly is lower than the efficiency of mapping quinoa reads onto the quinoa reference genome, we chose only quinoa accessions for CNV analysis. We also manually confirmed CNVs by comparing the differences in mapped reads between different samples using the BamView tool (ver 1.2.11) (Carver et al., 2013).

### Detection of single-nucleotide polymorphisms in candidate genes for *Chenopodium* accessions

To identify SNPs potentially related to the differences in salinity tolerance of the accessions, SNPs were called in each of the 219 candidate genes and from 2 kb upstream and downstream of the start and stop codons. SNP calling was performed with the mpileup function of SAMtools as previously described (Jarvis et al., 2017) with the exception that SNP positions were filtered for a minimum depth of four and a SNP allele frequency greater than 0.25. These SNPs were then further filtered for their presence or absence in the five most tolerant and five least tolerant accessions.

## Results

### Identification of differentially expressed genes involved in the salt-stress response

**Figure 2:**
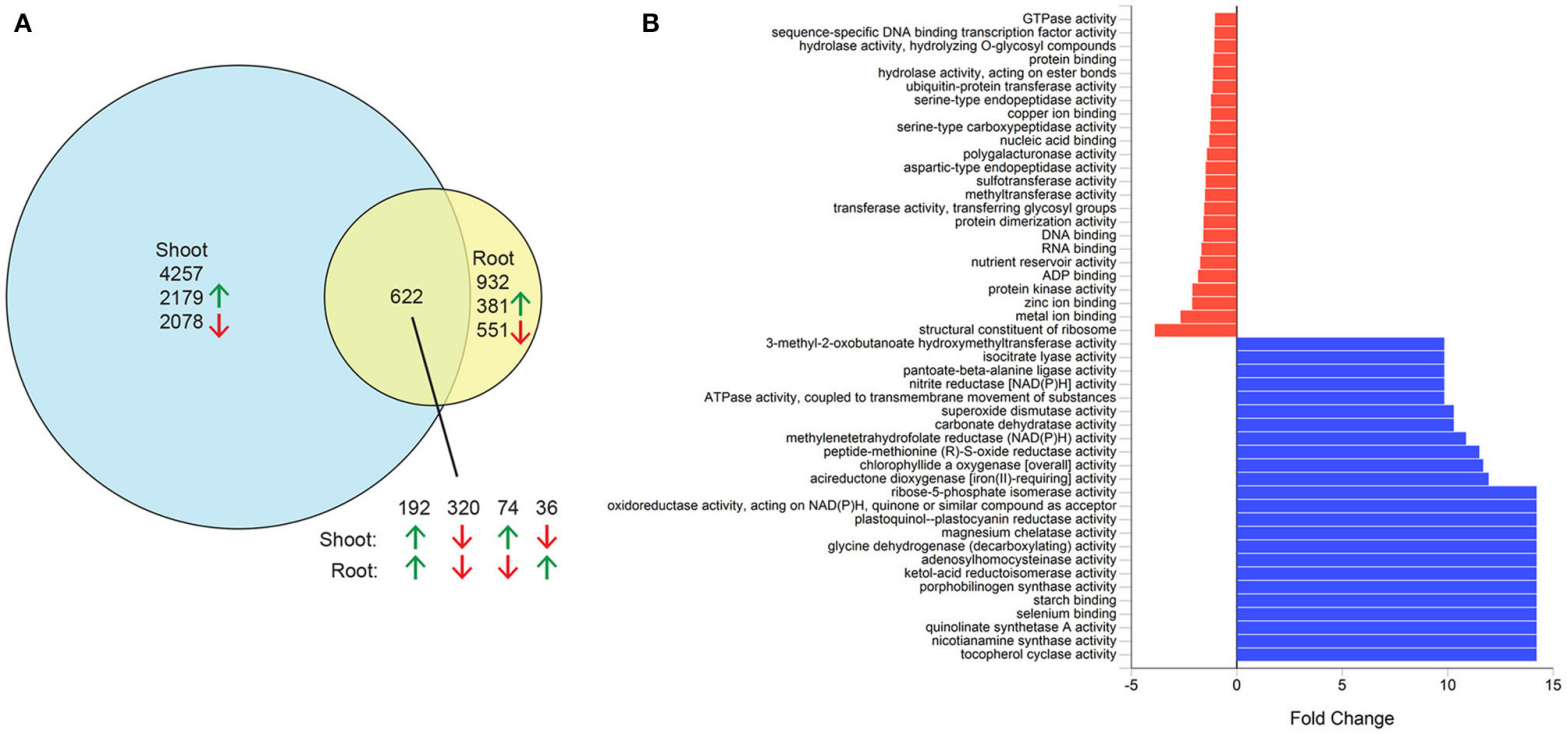
Differentially expressed genes in salt-stressed quinoa. Three-week-old hydroponically grown plants were grown under control conditions or treated with 300 mM NaCl for 1 week. Root and shoot samples were used for RNAseq analysis. **(A)** Numbers of differentially expressed genes in shoots, roots or both tissues. Green arrows, upregulated genes; red arrows, downregulated genes. **(B)** Fold change of select molecular function gene ontology terms of genes upregulated in shoots in response to salt. Blue bars, enriched gene ontology terms; red bars, depleted gene ontology terms.

As a first step to identify candidate genes involved in salinity tolerance in quinoa, we investigated genes that are differentially expressed in response to salinity stress in the reference genome quinoa accession PI 614886 (Jarvis et al., 2017). Shoots and roots of 4-week-old plants grown hydroponically in control conditions or treated with 300 mM NaCl for 7 days were used for RNA sequencing. Expression was detected from a total of 37,888 genes (85% of annotated genes), including 34,669 in the shoot and 36,126 in the root. Of these, 5,811 genes were differentially expressed between control and salinity treatments, including 4,257 genes differentially expressed only in shoots, 932 only in roots, and 622 in both shoots and roots (Figure 2A and Supplementary Data Sheet S1). In roots, more genes were downregulated than upregulated, whereas the number in shoots was similar. Analysis of GO terms assigned to these differentially expressed genes indicated an enrichment of genes involved in catalytic activity (Figure 2B and Supplementary Figure S1), suggesting that the expression of numerous enzymes increases in response to salinity stress.

### OrthoMCL analysis and TMD prediction provides putative membrane proteins distinct to quinoa

The recent release of the quinoa genome (Jarvis et al., 2017) represents the publication of the fourth genome sequence from the Amaranthaceae. The availability of these four genomes enables a comparative genomics approach to be used to identify potential gene candidates for salinity tolerance in quinoa. Given the relatively high salinity tolerance of quinoa and beet, genes that are unique to quinoa or that belong to gene families that have undergone expansion in quinoa and beet represent promising salinity tolerance candidates.

**Figure 3:**
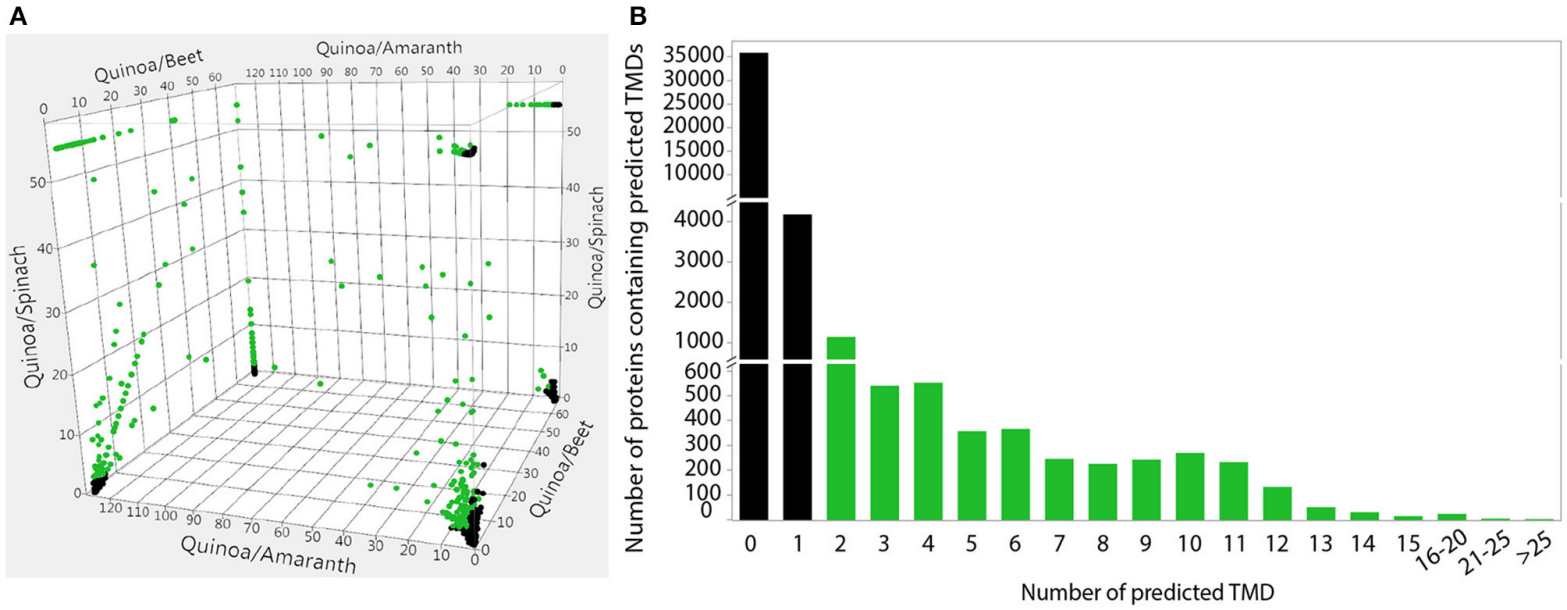
Identification of quinoa-distinct proteins that are unique to, or overrepresented in, quinoa (and beet) and prediction of transmembrane domains of quinoa proteins. **(A)** Gene family clusters were previously identified in the four sequenced Amaranthaceae species by OrthoMCL analysis (Jarvis et al., 2017). The ratio of genes from each species within these gene family clusters was plotted in three dimensions, with the 2-way ratios indicated on the axes. Clusters that contain an overrepresentation of quinoa (and beet) genes are highlighted in green and were used for subsequent analyses. **(B)** The number of transmembrane domains was predicted in the putative protein sequences of all annotated quinoa genes. Proteins with more than one predicted TMD, highlighted in green, were used in subsequent analyses. Predictions were performed using TMHMM Server v.2.0.

In addition to the 9,690 quinoa-specific genes that were previously identified (Jarvis et al., 2017), here we report the identification of an additional 10,125 genes (from 1,604 gene family clusters) that are overrepresented in quinoa and beet (Figure 3A and Supplementary Data Sheet S1). This represents a total of 19,815 quinoa candidate genes that we refer to as quinoa-distinct genes. Of these quinoa-distinct genes, 1,413 are differentially expressed in response to salt (Supplementary Data Sheet S1). We further restricted this list by focusing on proteins with transmembrane domains, because membrane proteins such as transporters have previously been shown to play crucial roles in salinity tolerance (e.g., as reviewed in Roy et al., 2014; Volkov, 2015). After selecting only genes that encode for proteins with more than one predicted TMD (Figure 3B and Supplementary Data Sheet S1), we identified 219 putative transmembrane genes that might be involved in salinity tolerance in quinoa.

### *Chenopodium* accessions show variation in salinity tolerance

**Figure 4:**
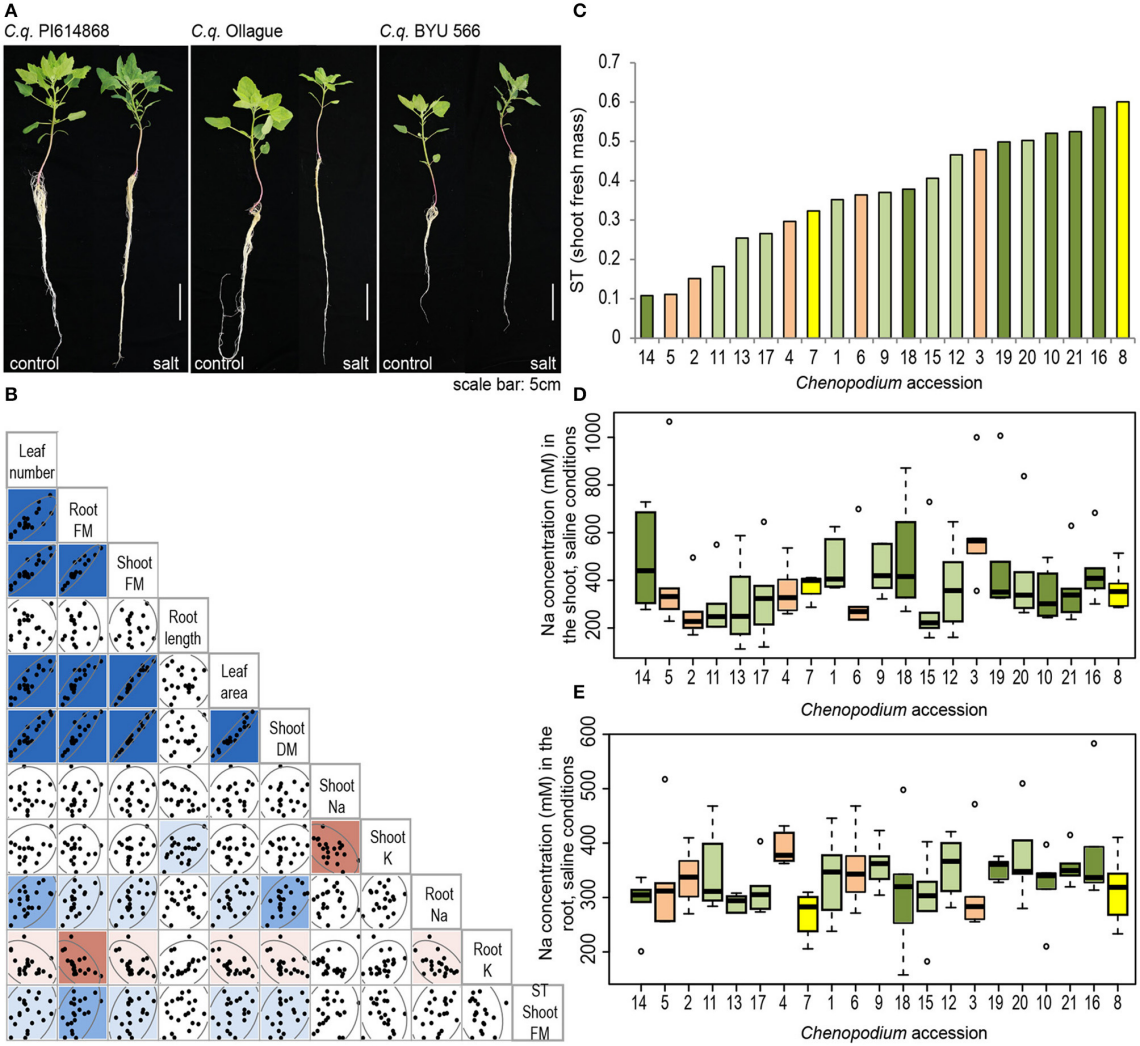
Contrasting growth phenotypes among 21 *Chenopodium* accessions. **(A)** Comparison of the phenotype of three selected accessions grown hydroponically under control conditions (left) or under treatment with 300 mM NaCl for 7 days (right). Photos were taken on the day of harvest. **(B)** Correlation analysis among traits hypothesized to contribute to salinity tolerance. Traits displayed for plants grown under saline conditions. FM, fresh mass; DM, dry mass; K, potassium concentration; Na, sodium concentration; ST, salinity tolerance index of shoot fresh mass. Dark blue indicates a strong positive correlation (*r* > 0.7), medium blue indicates a moderate positive correlation (*r* > 0.5) and light blue indicates a weak positive correlation (*r* > 0.3). Medium red indicates a moderate negative correlation (*r* < −0.5) and light red indicates a weak negative correlation (*r* < −0.3). **(C)** Salinity tolerance index (ST) of each *Chenopodium* accession. Orange bars, *C. berlandieri*; yellow bars, *C. hircinum*; light green bars, quinoa highland; dark green bars, quinoa coastal. **(D,E)** Sodium concentration in the **(D)** shoot and **(E)** root of salt-treated *Chenopodium* accessions (*n* = 6); statistical analyses were performed as described in the Methods section, (F statistic for **(D)** <0.0001 and for **(E)** <0.0001). Accessions are described in Table 1. In short: (1) *C. q*. 0654; (2) *C. b*. Huauzontle; (3) *C. b*. BYU 937; (4) *C. b*. BYU 803; (5) *C. b*. BYU 14104; (6) *C. b*. BYU 1314; (7) *C. h*. BYU 1101; (8) *C. h*. BYU 566; (9) *C. q*. CICA-17; (10) *C. q*. G-205; (11) *C. q*. Ollague; (12) *C. q*. Pasankalla; (13) *C. q*. Real; (14) *C. q*. Regalona; (15) *C. q*. Salcedo INIA; (16) *C. q*. Cherry Vanilla; (17) *C. q*. Chucapaca; (18) *C. q*. Ku-2; (19) *C. q*. Ames 22157; (20) *C. q*. Kurmi; (21) *C. q*. PI 614868.

To begin to determine whether these 219 preliminary candidate genes are involved in salinity tolerance in quinoa, we characterized several components of salinity tolerance of 21 *Chenopodium* accessions comprising 14 quinoa (8 highland and 6 coastal), 5 *C. berlandieri* and 2 *C. hircinum* accessions. These accessions were chosen because of the availability of their sequence information (Jarvis et al., 2017), which allowed us to integrate physiological data with genomic tools. We grew these accessions hydroponically under control conditions or with 300 mM NaCl and assessed shoot and root biomass (fresh and dry mass), leaf area, leaf number, root length and Na and K concentrations in the shoot and root. Different responses to salinity were observed (Figure 4A). For example after salt treatment, quinoa accession PI 614868 (Line 21; Figure 4A—left) maintained its shoot and root biomass, Ollague (Line 11; Figure 4A—middle) showed a substantial reduction in shoot and root biomass, and *C. hircinum* accession BYU 566 (Line 8; Figure 4A—right) maintained its biomass and had longer roots, but overall showed a lower shoot biomass in both control and salt conditions compared to these other two accessions.

Principal component analysis and pairwise correlation analyses suggest that under control and saline conditions the biomass related traits (root and shoot biomass, leaf area and leaf number) are strongly positively correlated (Figure 4B and Supplementary Figure S2). For ionic traits, principal component analyses and pairwise correlations deviate slightly. Both analyses suggest a strong negative correlation of shoot Na with shoot K, and a weak negative correlation of root Na with root K (Figure 4B and Supplementary Figure S2). Pairwise analyses suggest no correlation between shoot Na and root Na (Figure 4B). Additionally, mild positive correlations can be observed between root Na concentration and biomass related traits, as well as negative correlations between root K and biomass related traits (Figure 4B). Notably, there was no correlation between salinity tolerance (ST index) and any of the traits measured.

Salinity tolerance can be expressed as the maintenance of biomass under saline conditions (Negrão et al., 2016). The ST index based on fresh shoot mass shows that there is substantial variation between accessions, from 0.1 to 0.6 (Figure 4C). Growth of salt-sensitive lines, such as Regalona (Line 14, coastal) and Ollague (Line 11, highland), was reduced by 80–90% under salinity exposure compared to control conditions, while growth of the more salt-tolerant accessions, such as Cherry Vanilla (Line 16, coastal) and PI 614868 (Line 21, coastal), was only reduced by 40–50% (Figure 4C). Notably, two *C. berlandieri* accessions consistently have amongst the lowest ST indices (Lines 2 and 5), suggesting they are the least salt tolerant, while one accession of *C. hircinum* consistently has amongst the highest ST indices (Line 8), suggesting it has the highest salinity tolerance (Figure 4C and Supplementary Figure S3). The ranking of salt-tolerant and salt-sensitive accessions only changes marginally with calculations of salinity tolerance based on leaf area and leaf number, particularly for the top ranking and bottom ranking accessions (Supplementary Figure S3). Notably two *C. berlandieri* accessions (Lines 3 and 4) are top ranking for salt tolerance based on leaf number. For root fresh mass and root length the trend for salt tolerance index is not as consistent; however, it should be noted that one *C. hircinum* (Line 8) remains in the top three ranking accessions (Supplementary Figure S3).

A large variation in the concentration of Na was observed in the shoot, ranging from approximately 200 to over 500 mM Na in salt-treated accessions; the four accessions with the highest and lowest Na concentration are statistically different (Figure 4D). Variation in the Na concentration in the root was less evident with approximately 250–400 mM Na under salt treatment (Figure 4E). As expected, Na concentrations in control-treated accessions were very low in the root and shoot, ranging from approximately 5–20 mM Na (Supplementary Figures S4A,B). Root K concentrations did not change substantially under salinity treatment compared to control treatment (Supplementary Figures S4C,D), while in the shoot, some accessions displayed a significant increase in K under salt treatment compared to control (Supplementary Figure S4E). In the shoot, no significant difference in shoot K was found between control and salinity treatment or between the accessions (Supplementary Figure S4E).

### Copy number variation and single-nucleotide polymorphisms between tolerant and sensitive quinoa accessions

Having assessed the variation in salinity tolerance of these *Chenopodium* accessions, we next investigated variation in the 219 preliminary candidate genes in the most salt-tolerant and salt-sensitive accessions. For this, we evaluated differences in CNV and the presence of SNPs between tolerant and sensitive varieties.

According to the ST index based on fresh shoot mass, we selected the four top- and the four bottom-ranked quinoa accessions and performed pairwise comparisons for CNV from the 219 genes. 14 genes of interest were identified by the CNV analysis because they appear to have a gain or loss of mapped reads between the more and less tolerant accessions (Table 2). We found 8 of these are homologous to genes previously shown to participate in salinity stress responses. The rice homolog of *AUR62021463, OsGMST1*, has been shown to encode a monosaccharide transporter, with reduced expression conferring hypersensitivity to salinity stress in rice (Cao et al., 2011). In quinoa, expression of this gene increases 2-fold in shoots in response to salt stress (Table 2), suggesting that this transporter could contribute to salinity tolerance. *AUR62007451* has been annotated as a CYP75B1 (flavonoid monooxygenase), belonging to the diverse P450 gene family. There are at least 244 members of the P450 family that exhibit various functions in Arabidopsis. Little is known about the involvement of P450 proteins in salinity tolerance; however, knocking out a P450 monooxygenase, CYP709B3, causes Arabidopsis plants to become more salt-sensitive (Mao et al., 2013). *AUR62007451* has a 1.5-fold upregulation in the shoot in response to salt treatment (Table 2), consistent with the notion that increased expression may confer salinity tolerance. *AUR62004478* has been annotated as a cyclic nucleotide gated channel (CNGC), which has been suggested as a candidate to mediate Na^+^ entry into cells (Guo et al., 2008; Deinlein et al., 2014), and knock down of the Arabidopsis homolog appears to mediate salt sensitivity based on fresh biomass compared to wild type plants (Guo et al., 2008). AUR62034957 is annotated as an amino acid permease belonging to the AAP6 class. In Arabidopsis, AtAAP6 is a high affinity transporter of neutral and acidic amino acids, including proline (Rentsch et al., 1996), and is differentially regulated in response to abiotic stress in whole seedlings (Rentsch et al., 1996). Likewise, in quinoa, *AUR62034957* is up regulated in the shoot in response to salt stress. Given the role of proline as an osmoprotectant (Hayat et al., 2012), AUR62034957 may play a role in proline transport in response to salt stress in quinoa. A group of three quinoa genes, *AUR62011984, AUR62021522*, and *AUR62016440*, with homology to Arabidopsis sulfate transporters *SULTR1;1, SULTR3;4*, and *SULTR3;4*, respectively, were also identified. Expression of these quinoa genes is upregulated specifically in either shoot (*AUR62011984* and *AUR62021522*) or root (*AUR62016440*) tissues. Sulfate is an important component of plant abiotic stress tolerance and may help mediate salt stress tolerance via abscisic acid regulation of leaf stomatal conductance (Ernst et al., 2010) or by promoting synthesis of glutathione, which plays a role in cellular redox balance (Cao et al., 2014). Upregulation of sulfate transporters in the root may lead to increased sulfate uptake while increased expression in the shoot may result in increased transport of sulfate to specific aerial tissues. No direct association between salinity tolerance and the other 7 candidate genes identified by CNV analysis has been previously reported

**Table 2:** Candidate genes proposed to mediate salinity tolerance in quinoa.

**Gene ID**	**Annotation**	**Type of CNV**	**DE root**	**DE shoot**	**TMD**
AUR62006689	WAKL8 Wall-associated receptor kinase-like 8 (A.th.)	Loss in 8 comparisons (Kurmi and Cherry Vanilla vs. Regalona, Ollague, Real and Chucapaca)	−1.79	–	2
AUR62029668	Protein of unknown function	Loss in 8 comparisons (G-205 and Ames 22157 vs. Regalona, Ollague, Real and Chucapaca)	Inf	–	2
AUR62039756	At1g21890 WAT1-related protein At1g21890	Loss in 4 comparisons (Cherry Vanilla vs. Regalona, Ollague, Real and Chucapaca)	−2.31	–	9
AUR62021463	At1g67300 Probable plastidic glucose transporter 2	Gain in 6 comparisons (Kurmi, G-205 and Cherry Vanilla vs. Regalona and Chucapaca), loss in 2 comparisons (Ames 22157 vs. Ollague and Real)	–	1.04	10
AUR62007451	CYP75B1 Flavonoid 3'-monooxygenase (A.th.)	Gain in 4 comparisons (Kurmi, G-205, Ames 22157 and Cherry Vanilla vs. Regalona)	–	0.63	2
AUR62039871	psbD Photosystem II D2 protein (*Lobularia maritima*)	Gain in 4 comparisons (Cherry Vanilla vs. Regalona, Ollague and Real) and (Ames 22157 vs. Regalona)	–	0.97	5
AUR62043781	CER1: Protein ECERIFERUM 1 (A.th.)	Gain in 6 comparisons (Kurmi and G-205 vs. Ollague and Regalona), (Kurmi vs. Real), (Ames 22157 vs. Ollague)	–	−0.90	6
AUR62043583	Unknown function	SNP that is correlated with reduced salinity tolerance of *C. berlandieri* relative to *C. hircinum* and quinoa	Inf	–	4
AUR62034957	AAP6 Amino acid permease 6 (A.th.)	Loss in 6 comparisons (Kurmi vs. Real), Cherry Vanilla vs. Regalona, Ollague, Real and Chucapaca), (Ames 22157 vs. Ollague), gain in 2 comparisons (G-205 vs. Regalona and Chucapaca)	–	0.81	2
AUR62011984	SULTR1;1 Sulfate transporter 1.1 (A.th.)	Loss in 4 comparisons (Cherry Vanilla vs. Regalona, Ollague, Real and Chucapaca)	–	1.44	2
AUR62021522	SULTR3;4 Probable sulfate transporter 3.4 (A.th.)	Loss in 6 comparisons (Cherry Vanilla vs. Regalona, Ollague, Real and Chucapaca), (Ames 22157 vs. Ollague and Real), gain in 2 comparisons (G-205 vs. Regalona and Chucapaca)	–	0.80	9
AUR62016440	SULTR3;4 Probable sulfate transporter 3.4 (A.th.)	Loss in 4 comparisons (Cherry Vanilla and Ames 22157 vs. Ollague and Real)	2.63	–	10
AUR62004478	CNGC7 Putative cyclic nucleotide-gated ion channel 7 (A.th.)	Loss in 4 comparisons (Cherry Vanilla vs. Regalona, Ollague, Real and Chucapaca)	−1.37	−0.77	2
AUR62002768	DTX14 Protein DETOXIFICATION 14 (A.th.)	Gain in 4 comparisons (Kurmi, G-205, Ames 22157 and Cherry Vanilla vs. Real)	–	0.91	5
AUR62041961	TMK1 Receptor protein kinase 1 (A.th.)	Gain in 4 comparisons (Kurmi, G-205, Ames 22157 and Cherry Vanilla vs. Chucapaca)	–	−2.37	6

*DE, differential expression (log_2_); TMD, transmembrane domains; inf, infinite increase (no reads for control condition); A.th., Arabidopsis thaliana*.

In addition to CNV analysis, SNPs were called in the 219 selected genes and the differential presence/absence of these SNPs was examined in the five top- and the five bottom-ranked *Chenopodium* accessions, according to the ST index based on fresh shoot mass. Of the 230 SNPs identified as being differentially present/absent in the most salt tolerant lines, 6 of these were located in the first exon of *AUR62043583* (Supplementary Data Sheet S2). While this gene is annotated as a protein of unknown function, comparisons with sequence databases indicate that the gene encodes an ankyrin repeat-containing protein. *AUR62043583* might encode for the same ankyrin repeat-containing protein to which the Shaker-like K^+^ channels belong (Becerra et al., 2004), making this a candidate potentially involved in maintaining ion homeostasis and therefore perhaps facilitating salinity tolerance. This gene, as well as the 14 candidate genes identified from the CNV analysis, represents promising targets for future functional studies to determine their role in salinity tolerance in quinoa and related species.

## Discussion

In this study, we describe the integration of physiological measurements of plant growth under salt stress with several complementary bioinformatics approaches to identify promising candidate genes. First, we identified genes that are differentially expressed in response to salinity stress in the reference quinoa accession PI 614886. We then selected a sub-set of these differentially expressed genes that are unique to, or overrepresented in, quinoa (and those that are also overrepresented in beet, which has a comparable salt tolerance to quinoa). To identify putative transmembrane proteins, we only focused on genes that encode proteins with more than one TMD. This set of 219 genes represent putative membrane-bound proteins involved in salinity tolerance in quinoa, although we recognize that other proteins, including soluble proteins such as kinases, likely also play an important role in salinity tolerance and should therefore not be overlooked (Supplementary Data Sheet S1) (Roy et al., 2013).

To determine whether these 219 genes contribute to variation in salinity tolerance of quinoa, we screened 21 *Chenopodium* accessions (14 quinoa, 5 *C. berlandieri*, and 2 *C. hircinum*) for salinity tolerance and observed phenotypic variation among all measured traits, including the ST index and Na content in the shoot and the root. We then searched for SNPs and CNVs in these genes in the most and least salt-tolerance accessions. This approach enabled us to identify 15 candidate genes that putatively underlie variation for salinity tolerance in these accessions. Future experiments will aim to validate the role of these candidate genes in salinity tolerance, although, to date, this has been difficult as stable transformations of quinoa have not been reported yet. However, viruses that may induce gene silencing have previously been amplified using quinoa as a host plant (Kotoda and Wada, 2005); hence, this system might also be used in the future for functional studies, in which expression of quinoa genes is downregulated.

Several mechanisms regulate salinity tolerance in plants, including Na exclusion, which refers to the minimization of Na accumulation in the shoot, and tissue tolerance, which refers to the compartmentalization of Na. If one of these mechanisms were dominant in our screened *Chenopodium* accessions, a strong correlation would be seen between ST (e.g., maintenance of biomass) and the concentration of Na in the shoot. This has been shown, for instance, in tetraploid wheat when grown at high salinity stress (Munns and James, 2003). It has been argued that quinoa's high salinity tolerance is attributed to tight control of ion homeostasis either by excluding Na from the shoot, using it for osmotic adjustment, or by sequestering it into the vacuole (Adolf et al., 2013); however, we did not see a correlation where accessions that accumulate higher (or lower) amounts of Na or K have higher salinity tolerance. It is likely that other mechanisms are involved that explain the variation in salinity tolerance in these quinoa accessions.

It is notable that treatment with 300 mM NaCl had no significant effect on some accessions, such that some ST indices were calculated to be greater than one. This indicates that these accessions not only maintained their growth under saline conditions, but that they exceeded their growth under control conditions. For example, *C. hircinum* BYU 566 (Line 8) performed better under saline than control conditions, although accumulation of Na was neither particularly high nor low in its roots or shoots (compared to the other accessions). This is consistent with observations of some quinoa accessions in which growth was stimulated at moderate salinity (100–200 mM NaCl) (Jacobsen et al., 2003; Gomez-Pando et al., 2010).

For some accessions, K concentrations under saline conditions were higher than under control conditions, particularly in the shoot, but these were not correlated with salinity tolerance. This has also been observed in a previous study investigating the two quinoa accessions Titicaca and Utusaya (Adolf et al., 2012). It has been hypothesized previously that not only low cytosolic Na concentrations, but also the maintenance of high cytosolic K concentrations within a plant are important for salinity tolerance (Shabala and Cuin, 2008). The high salinity tolerance of quinoa may be attributed in part to its ability to maintain K homeostasis and therefore maintain enzymatic functions important for plant growth (Munns and Tester, 2008). A candidate gene that we identified, *AUR62043583*, shares homology with ankyrin repeat proteins and is related to shaker-like K^+^ channels (Becerra et al., 2004). Therefore, it is possible that this gene plays a role in facilitating high K concentrations in the shoot under salinity stress, thereby helping the plant maintain a favorable Na to K ratio.

The large phenotypic variation that was observed among the 21 accessions might represent a basis for genetic studies to identify loci contributing to salinity tolerance (such as genome-wide association studies). For this, a larger panel of accessions is needed, and based on this study we would suggest including a larger collection of *C. hircinum*, because *C. hircinum* BYU 556 exhibited the highest salinity tolerance for most traits measured, making it ideal for identifying loci contributing to salinity tolerance. Only two *C. hircinum* accessions were available for inclusion in our analysis and thus it is prudent that more accessions be added for further salinity tolerance and genetic studies. To date, the salinity tolerance of *C. hircinum* remains poorly studied, as evidenced by the lack of available literature.

We also suggest that both coastal and highland varieties of quinoa should be used in phenotypic studies for abiotic stress tolerance. Based on previous SNP analyses, coastal and highland accessions form two distinct clades (Jarvis et al., 2017), suggesting that these accessions might also cluster together for salinity tolerance traits. However, based on this study, it appears that coastal and highland accessions do not cluster together for salinity stress tolerance. For example, we observed that for some traits (e.g., shoot fresh mass) a coastal accession was the most salt tolerant, and for other traits (e.g., root fresh mass and leaf area) a highland accession was the most salt tolerant. This inconsistency has also been shown in a previous study examining three accessions, in which the highland (Salares) ecotype appeared to perform better under salinity stress compared to two coastal accessions in some traits measured, but not in others (Ruiz et al., 2016).

## Data accessibility

RNAseq reads were deposited at NCBI under the BioProject code PRJNA306026, Experiment codes SRX2830838–SRX2830843, SRX2830856–SRX2830858, SRX2830862, and SRX2830863.

## Author contributions

SS, DL, and DJ performed the experiments and analyzed the data. SS led the physiological characterization of plants and drafted the manuscript. DL led the OrthoMCL and SNP polymorphism analyses. RR led the CNV analyses. MT contributed to the original concept. DJ lead RNAseq analyses. All authors read and approved the final manuscript.

### Conflict of interest statement

The authors declare that the research was conducted in the absence of any commercial or financial relationships that could be construed as a potential conflict of interest.
