# Supplementary material for: Identification of Putative Transmembrane Proteins Involved in Salinity Tolerance in Chenopodium quinoa by Integrating Physiological Data, RNAseq, and SNP Analyses
Source: Front Plant Sci. 2017 Jun 21;8:1023. doi: 10.3389/fpls.2017.01023 (PMC5478719; doi:10.3389/fpls.2017.01023)
Supplement: Supplementary file 3 [file Image1.PDF]

Supplementary Material

Identification of putative transmembrane proteins involved in salinity tolerance in *Chenopodium quinoa* by integrating physiological data, RNAseq and SNP analyses

Sandra M. Schmöckel[1,†], Damien J. Lightfoot[1,2,†], Rozaimi Razali[1,3], Mark Tester[1], David E. Jarvis[1,*]

*** Correspondence:** David E. Jarvis: david.jarvis@kaust.edu.sa

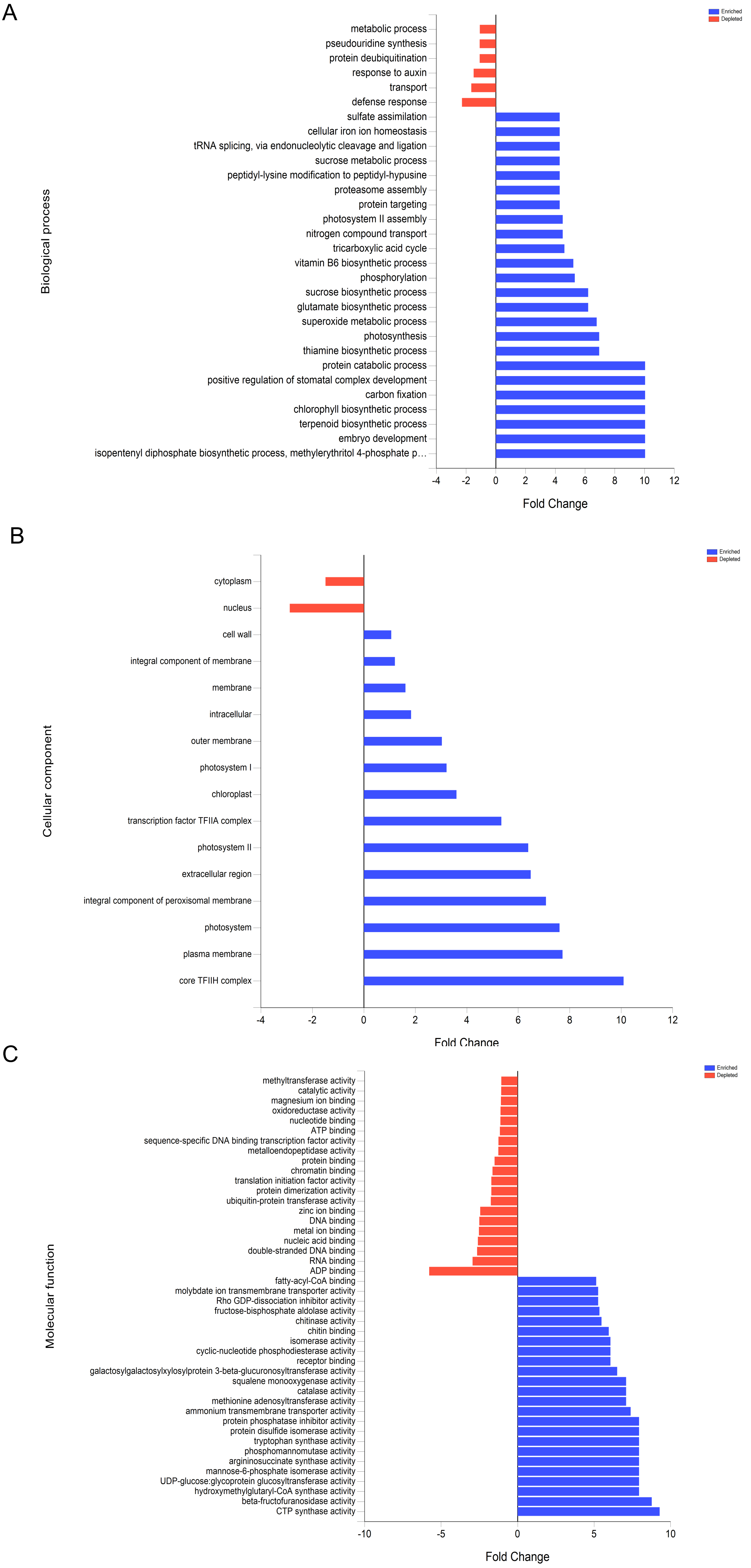
A
Enriched
Depleted
Biological process
metabolic process
pseudouridine synthesis
protein deubiquitination
response to auxin
transport
defense response
sulfate assimilation
cellular iron ion homeostasis
tRNA splicing, via endonucleolytic cleavage and ligation
sucrose metabolic process
peptidyl-lysine modification to peptidyl-hypusine
proteasome assembly
protein targeting
photosystem II assembly
nitrogen compound transport
tricarboxylic acid cycle
vitamin B6 biosynthetic process
phosphorylation
sucrose biosynthetic process
glutamate biosynthetic process
superoxide metabolic process
photosynthesis
thiamine biosynthetic process
protein catabolic process
positive regulation of stomatal complex development
carbon fixation
chlorophyll biosynthetic process
terpenoid biosynthetic process
embryo development
isopentenyl diphosphate biosynthetic process, methylerythritol 4-phosphate p…
-4
-2
0
2
4
6
8
10
12
Fold Change
B
Enriched
Depleted
Cellular component
cytoplasm
nucleus
cell wall
integral component of membrane
membrane
intracellular
outer membrane
photosystem I
chloroplast
transcription factor TFIIA complex
photosystem II
extracellular region
integral component of peroxisomal membrane
photosystem
plasma membrane
core TFIIH complex
-4
-2
0
2
4
6
8
10
12
Fold Change
C
Enriched
Depleted
Molecular function
methyltransferase activity
catalytic activity
magnesium ion binding
oxidoreductase activity
nucleotide binding
ATP binding
sequence-specific DNA binding transcription factor activity
metalloendopeptidase activity
protein binding
chromatin binding
translation initiation factor activity
protein dimerization activity
ubiquitin-protein transferase activity
zinc ion binding
DNA binding
metal ion binding
nucleic acid binding
double-stranded DNA binding
RNA binding
ADP binding
fatty-acyl-CoA binding
molybdate ion transmembrane transporter activity
Rho GDP-dissociation inhibitor activity
fructose-bisphosphate aldolase activity
chitinase activity
chitin binding
isomerase activity
cyclic-nucleotide phosphodiesterase activity
receptor binding
galactosylgalactosylxylosylprotein 3-beta-glucuronosyltransferase activity
squalene monooxygenase activity
catalase activity
methionine adenosyltransferase activity
ammonium transmembrane transporter activity
protein phosphatase inhibitor activity
protein disulfide isomerase activity
tryptophan synthase activity
phosphomannomutase activity
argininosuccinate synthase activity
mannose-6-phosphate isomerase activity
UDP-glucose:glycoprotein glucosyltransferase activity
hydroxymethylglutaryl-CoA synthase activity
beta-fructofuranosidase activity
CTP synthase activity
-10
-5
0
5
10
Fold Change

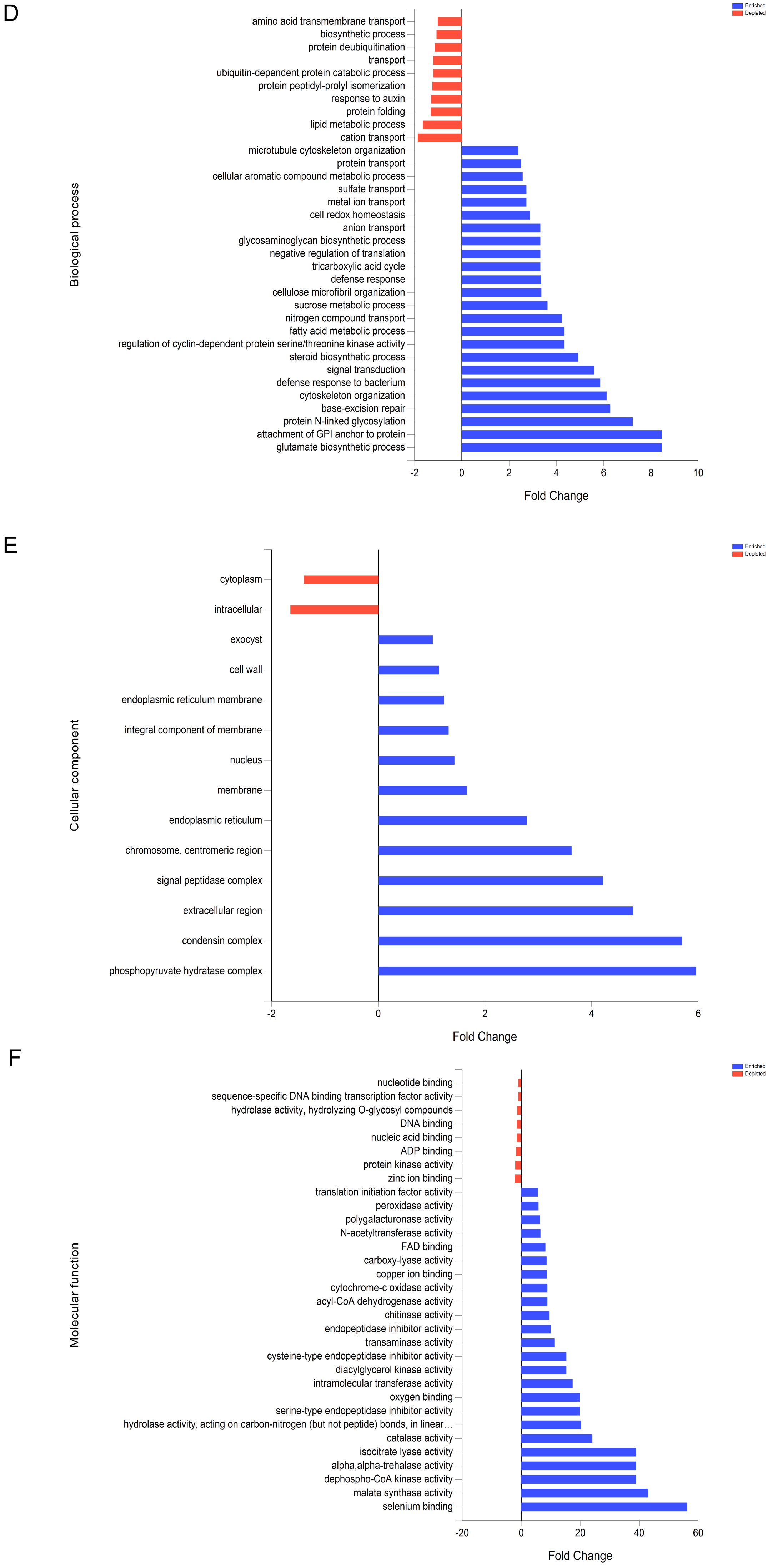
D
Enriched
Depleted
Biological process
amino acid transmembrane transport
biosynthetic process
protein deubiquitination
transport
ubiquitin-dependent protein catabolic process
protein peptidyl-prolyl isomerization
response to auxin
protein folding
lipid metabolic process
cation transport
microtubule cytoskeleton organization
protein transport
cellular aromatic compound metabolic process
sulfate transport
metal ion transport
cell redox homeostasis
anion transport
glycosaminoglycan biosynthetic process
negative regulation of translation
tricarboxylic acid cycle
defense response
cellulose microfibril organization
sucrose metabolic process
nitrogen compound transport
fatty acid metabolic process
regulation of cyclin-dependent protein serine/threonine kinase activity
steroid biosynthetic process
signal transduction
defense response to bacterium
cytoskeleton organization
base-excision repair
protein N-linked glycosylation
attachment of GPI anchor to protein
glutamate biosynthetic process
-2
0
2
4
6
8
10
Fold Change
E
Enriched
Depleted
Cellular component
cytoplasm
intracellular
exocyst
cell wall
endoplasmic reticulum membrane
integral component of membrane
nucleus
membrane
endoplasmic reticulum
chromosome, centromeric region
signal peptidase complex
extracellular region
condensin complex
phosphopyruvate hydratase complex
-2
0
2
4
6
Fold Change
F
Enriched
Depleted
Molecular function
nucleotide binding
sequence-specific DNA binding transcription factor activity
hydrolase activity, hydrolyzing O-glycosyl compounds
DNA binding
nucleic acid binding
ADP binding
protein kinase activity
zinc ion binding
translation initiation factor activity
peroxidase activity
polygalacturonase activity
N-acetyltransferase activity
FAD binding
carboxy-lyase activity
copper ion binding
cytochrome-c oxidase activity
acyl-CoA dehydrogenase activity
chitinase activity
endopeptidase inhibitor activity
transaminase activity
cysteine-type endopeptidase inhibitor activity
diacylglycerol kinase activity
intramolecular transferase activity
oxygen binding
serine-type endopeptidase inhibitor activity
hydrolase activity, acting on carbon-nitrogen (but not peptide) bonds, in linear…
catalase activity
isocitrate lyase activity
alpha,alpha-trehalase activity
dephospho-CoA kinase activity
malate synthase activity
selenium binding
-20
0
20
40
60
Fold Change

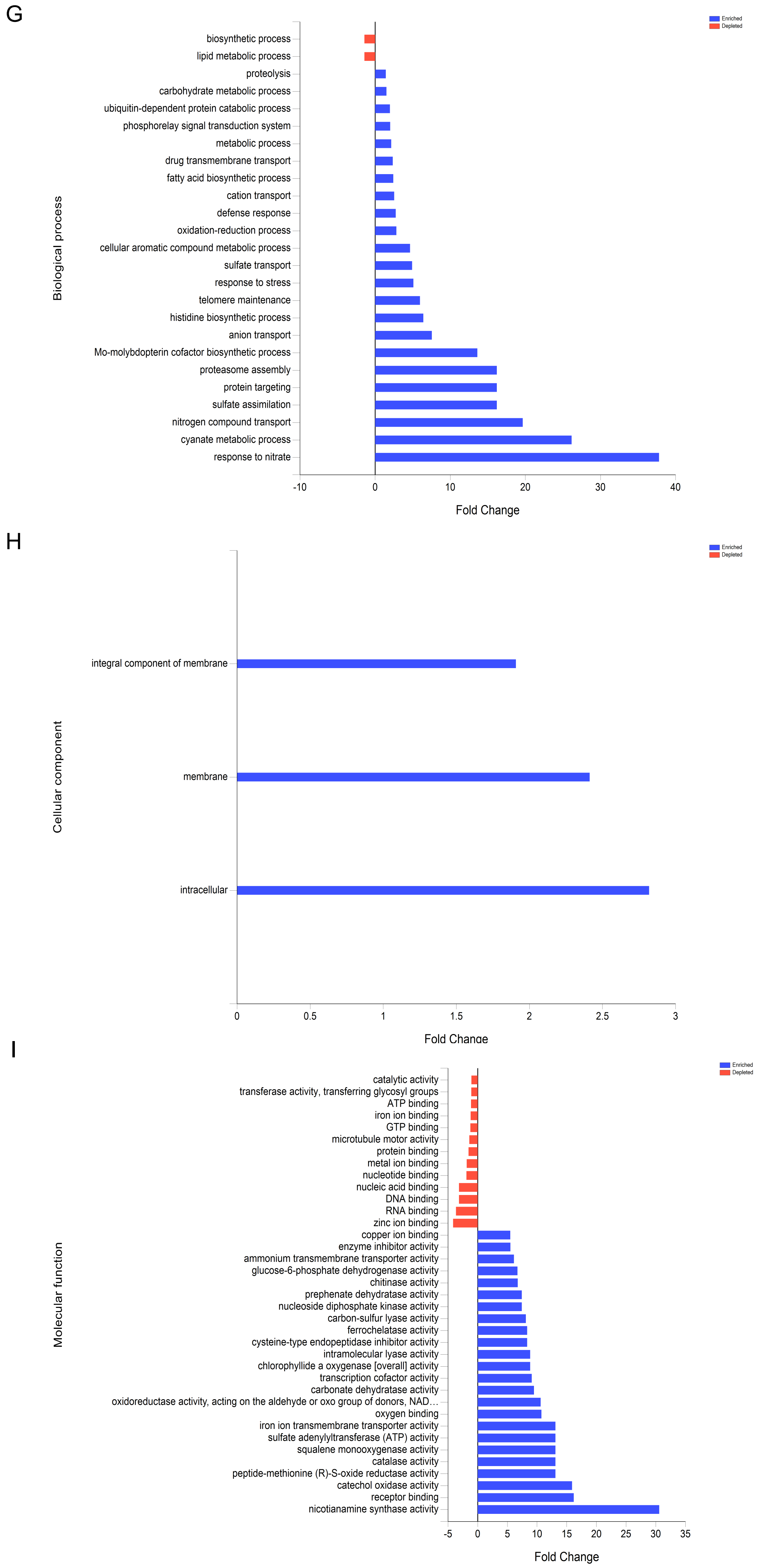
G
Enriched
Depleted
Biological process
biosynthetic process
lipid metabolic process
proteolysis
carbohydrate metabolic process
ubiquitin-dependent protein catabolic process
phosphorelay signal transduction system
metabolic process
drug transmembrane transport
fatty acid biosynthetic process
cation transport
defense response
oxidation-reduction process
cellular aromatic compound metabolic process
sulfate transport
response to stress
telomere maintenance
histidine biosynthetic process
anion transport
Mo-molybdopterin cofactor biosynthetic process
proteasome assembly
protein targeting
sulfate assimilation
nitrogen compound transport
cyanate metabolic process
response to nitrate
-10
0
10
20
30
40
Fold Change
H
Enriched
Depleted
Cellular component
integral component of membrane
membrane
intracellular
0
0.5
1
1.5
2
2.5
3
Fold Change
I
Enriched
Depleted
Molecular function
catalytic activity
transferase activity, transferring glycosyl groups
ATP binding
iron ion binding
GTP binding
microtubule motor activity
protein binding
metal ion binding
nucleotide binding
nucleic acid binding
DNA binding
RNA binding
zinc ion binding
copper ion binding
enzyme inhibitor activity
ammonium transmembrane transporter activity
glucose-6-phosphate dehydrogenase activity
chitinase activity
prephenate dehydratase activity
nucleoside diphosphate kinase activity
carbon-sulfur lyase activity
ferrochelatase activity
cysteine-type endopeptidase inhibitor activity
intramolecular lyase activity
chlorophyllide a oxygenase [overall] activity
transcription cofactor activity
carbonate dehydratase activity
oxidoreductase activity, acting on the aldehyde or oxo group of donors, NAD…
oxygen binding
iron ion transmembrane transporter activity
sulfate adenylyltransferase (ATP) activity
squalene monooxygenase activity
catalase activity
peptide-methionine (R)-S-oxide reductase activity
catechol oxidase activity
receptor binding
nicotianamine synthase activity
-5
0
5
10
15
20
25
30
35
Fold Change

J

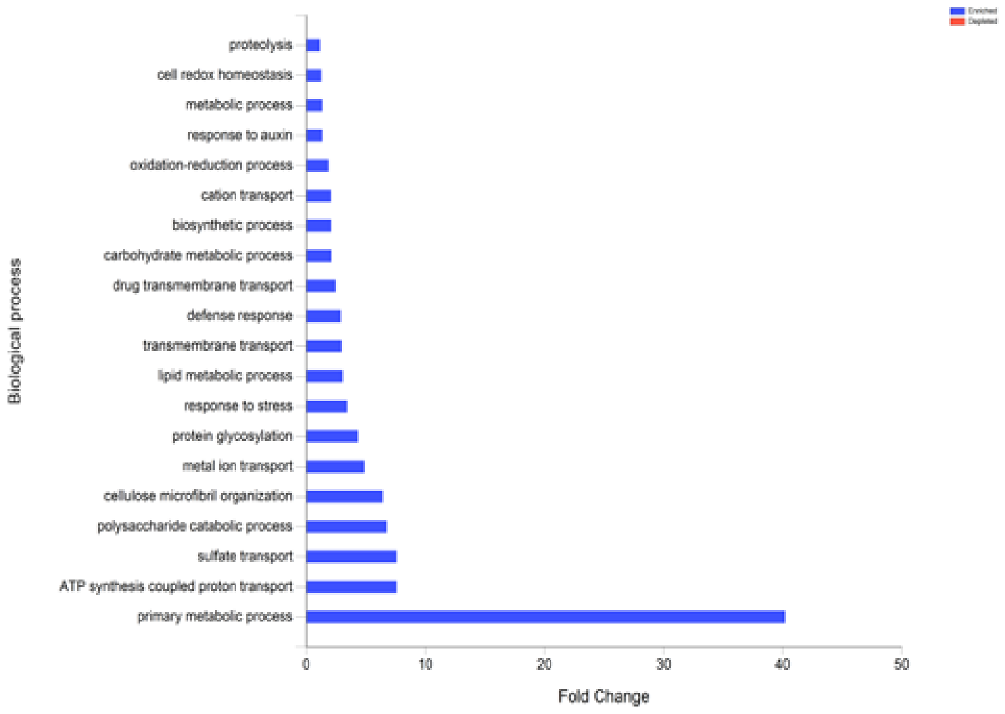

K

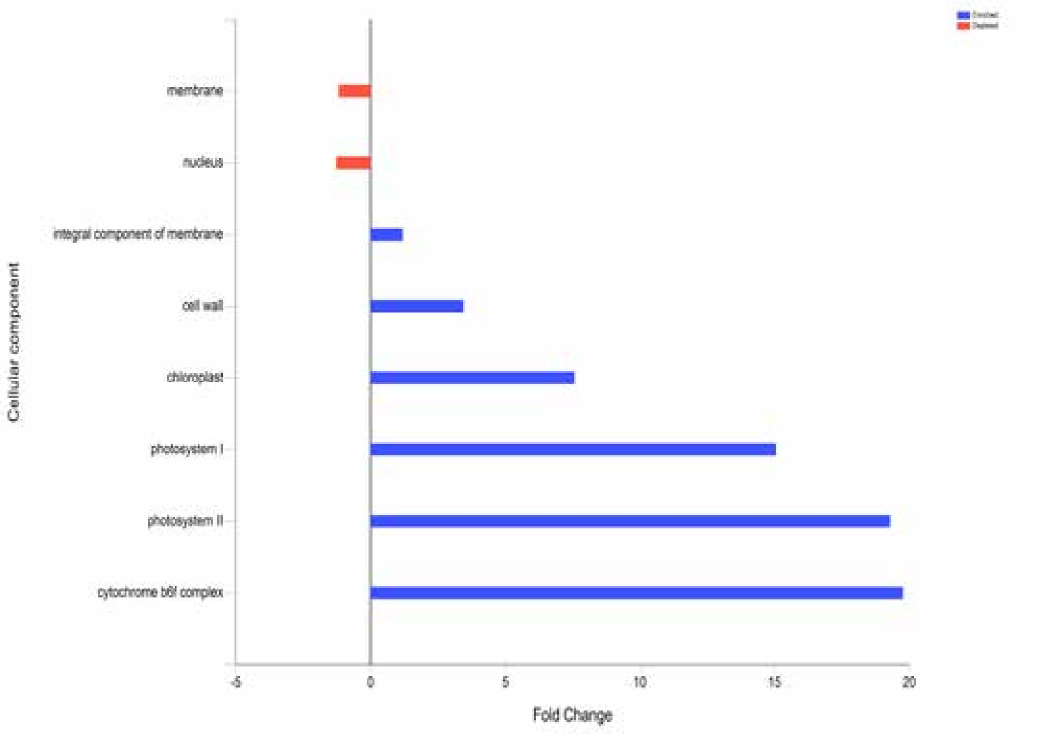

Supplementary Figure S1: Fold change of select gene ontology terms of genes differentially expressed in response to salt stress. Three-wk-old hydroponically grown plants were grown under control conditions or treated with 300 mM NaCl for 1 wk. Root and shoot samples were used for RNAseq analysis. Fold change of select biological process (A) and cellular component (B) gene ontology terms of genes upregulated in shoots in response to salt. Fold change of select molecular function (C, F, I), biological process (D, G, J) and cellular component (E, H, K) gene ontology terms of genes downregulated in shoots (C, D, E), upregulated in roots (F, G, H), or downregulated in roots (I, J, K) in response to salt. Blue bars, enriched gene ontology terms; red bars, depleted gene ontology terms.

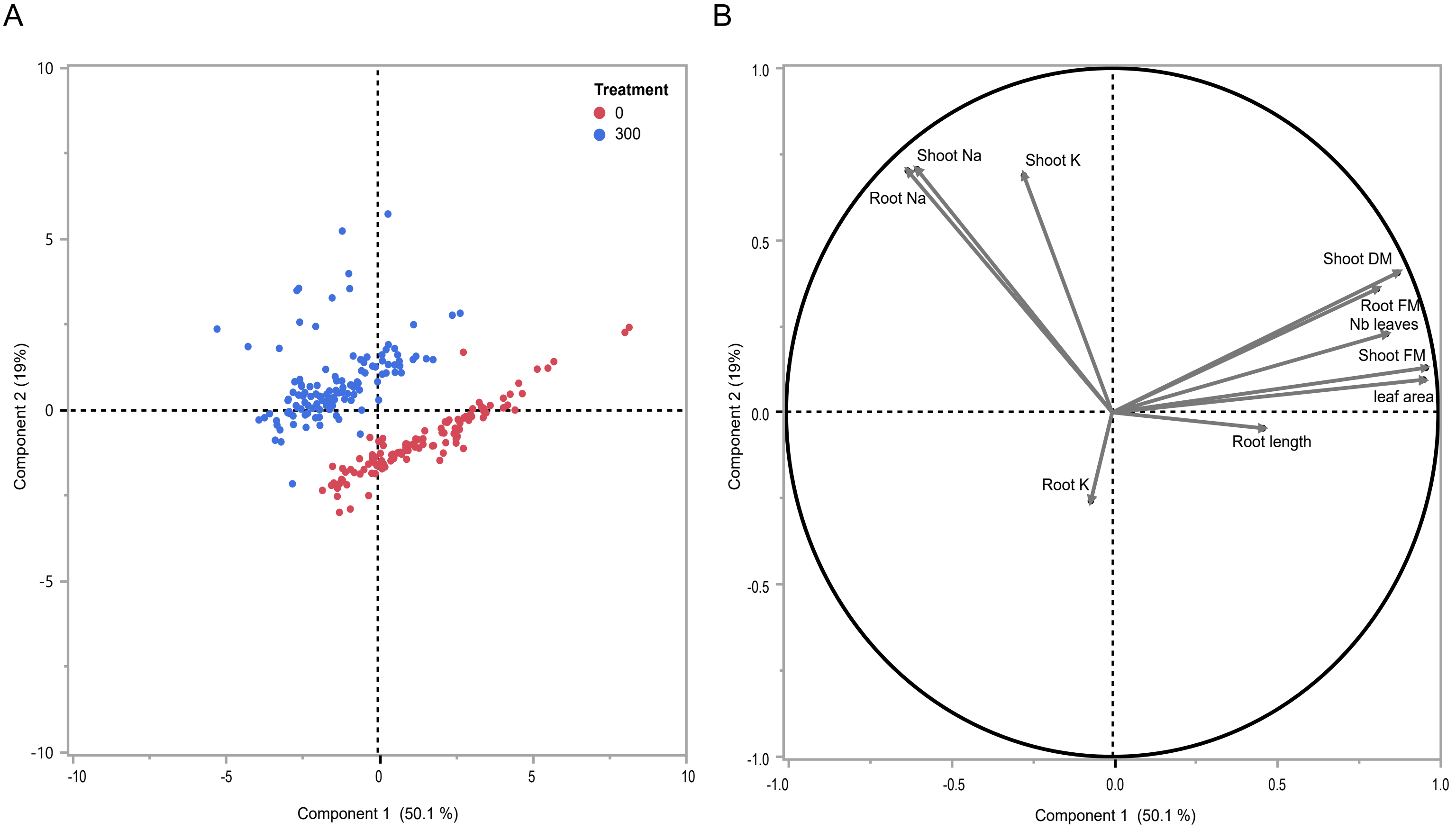

Supplementary Figure S2: Principal component analysis for traits hypothesized to contribute to salinity tolerance. (A) Scatter plot of PCA analysis with control (red) and salt treated (blue) *Chenopodium* accessions. Plotted points represent the average value for each measured trait for each accession. (B) Arrows indicate PCA analysis for traits indicated. All statistical analyses were performed in JMP.

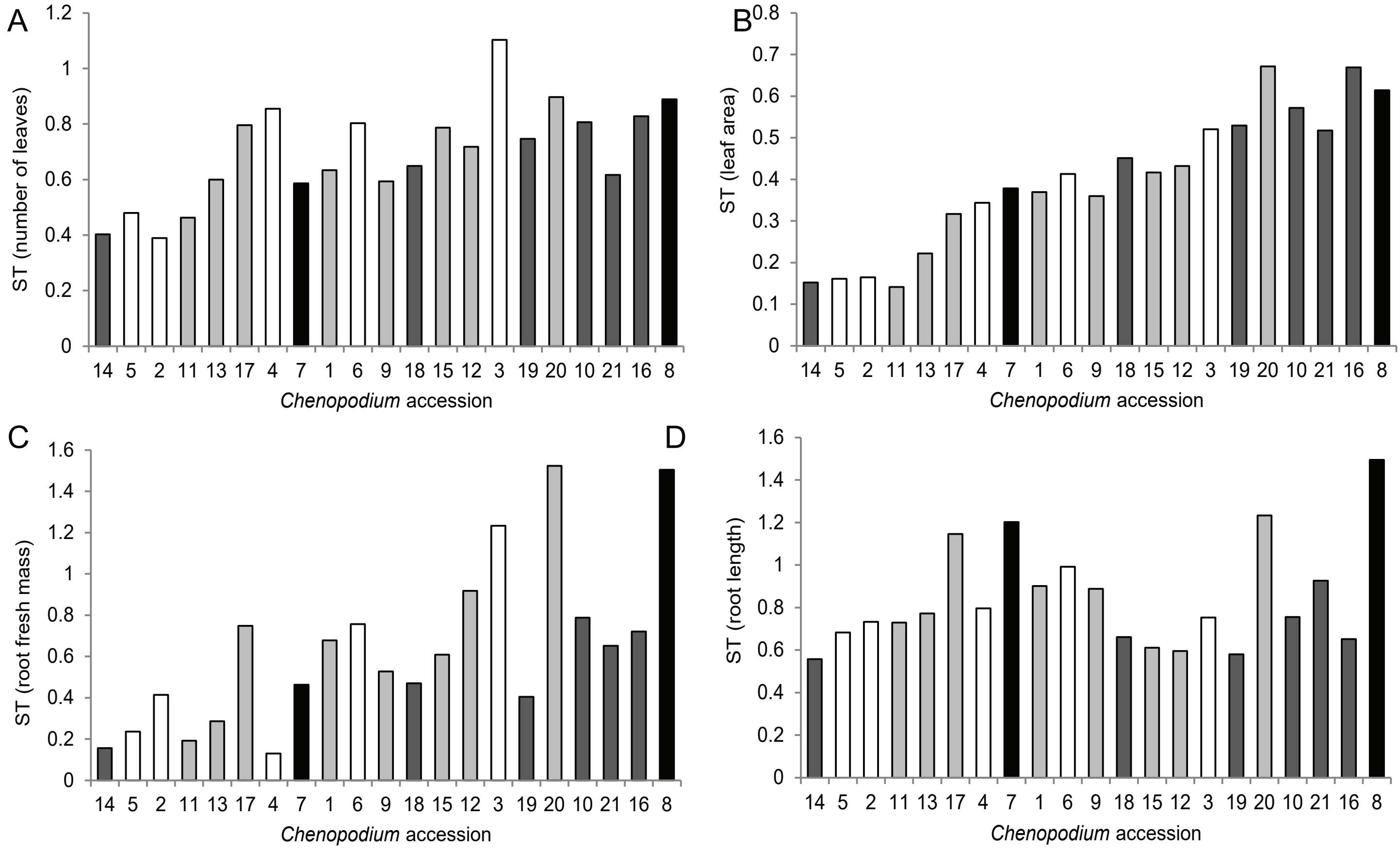

Supplementary Figure S3: Comparison of salt tolerance (ST) of different *Chenopodium* accessions. ST was calculated for (A) number of leaves, (B) leaf area, (C) fresh root mass and (D) root length. ST was calculated as the ratio of the trait during the salinity treatment compared to the control treatment. Accessions are described in Table 1. In short: (1) *C. q*. 0654; (2) *C. b*. PI 568156; (3) *C. b*. BYU 937; (4) *C. b*. PI 666279; (5) *C. b.* Ames 33013; (6) *C. b.* BYU 1314; (7) *C. h.* BYU 1101; (8) *C. h.* BYU 566; (9) *C. q.* CICA-17; (10) *C. q.* G-205-95DK; (11) *C. q.* Ollague; (12) *C. q.* Pasankalla; (13) *C. q.* Real; (14) *C. q.* Regalona; (15) *C. q.* Salcedo INIA; (16) *C. q.* Cherry Vanilla; (17) *C. q.* Ku-2; (18) *C. q.* Chucapaca (19) *C. q.* PI 634921; (20) *C. q.* Kurmi; (21) *C. q.* PI 614868.

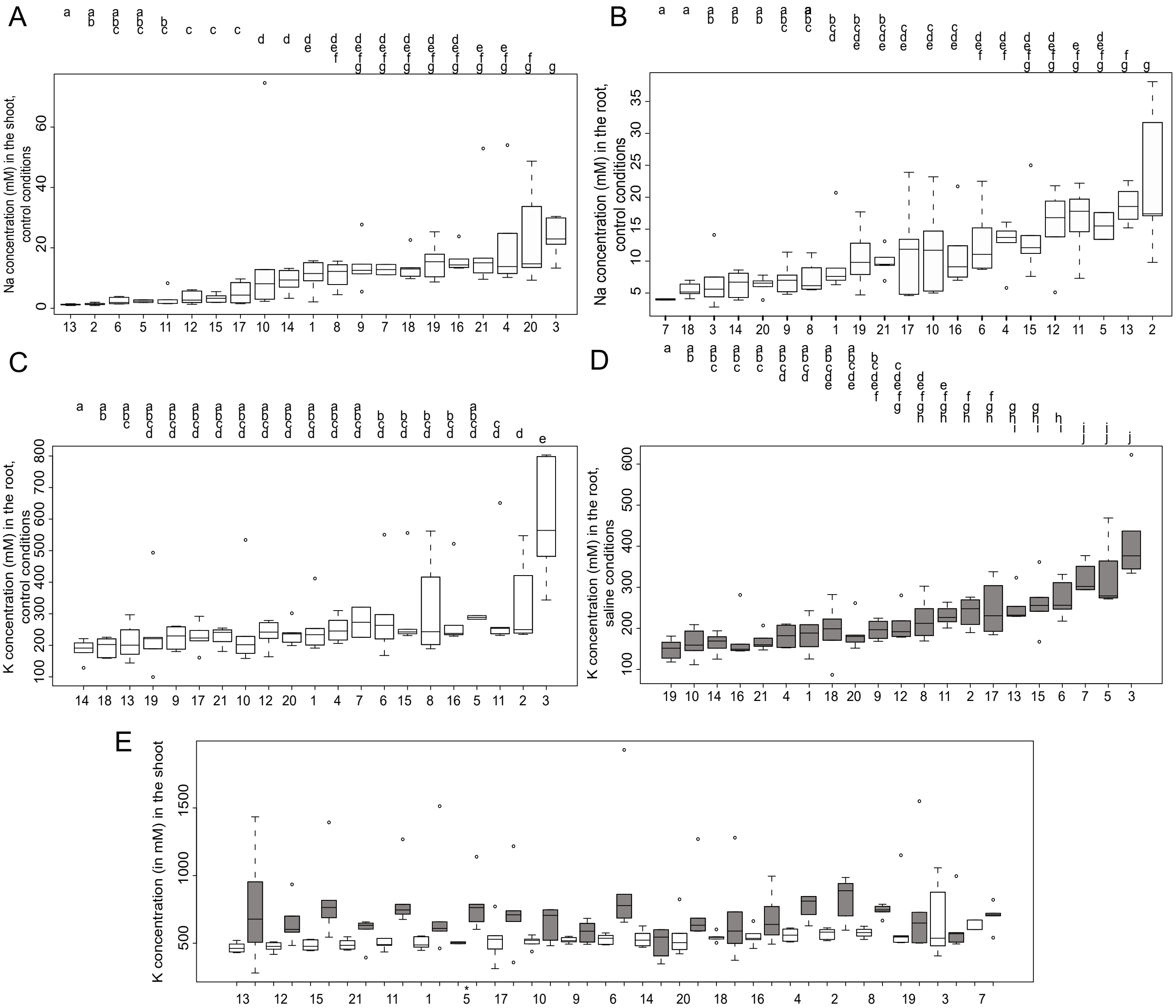

Supplementary Figure S4: Na and K distribution in salt stressed *Chenopodium* accessions. (A) Na concentration in the shoot and (B) root of control treated *Chenopodium* accessions. (C) K concentration in the root of control and (D) salt treated *Chenopodium* accessions. (E) K concentration in the shoot of control (in light grey) and salt (in dark grey) treated *Chenopodium* accessions; n=6, statistical analyses were performed as described in the methods section. F-statistic for (A), (B), (C) and (D) accession <0.0001, and for (E) accession 0.911 and treatment 0.062; levels not connected by the same letter are significantly different, $P \leq 0.05$. (Different layouts were chosen as the result of the fitted model suggesting in (E) no significant differences between accessions and treatment.)
