# Supplementary material for: Identification of Putative Transmembrane Proteins Involved in Salinity Tolerance in Chenopodium quinoa by Integrating Physiological Data, RNAseq, and SNP Analyses
Source: Front Plant Sci. 2017 Jun 21;8:1023. doi: 10.3389/fpls.2017.01023 (PMC5478719; doi:10.3389/fpls.2017.01023)
Supplement: Supplementary file 4 [file Image2.pdf]

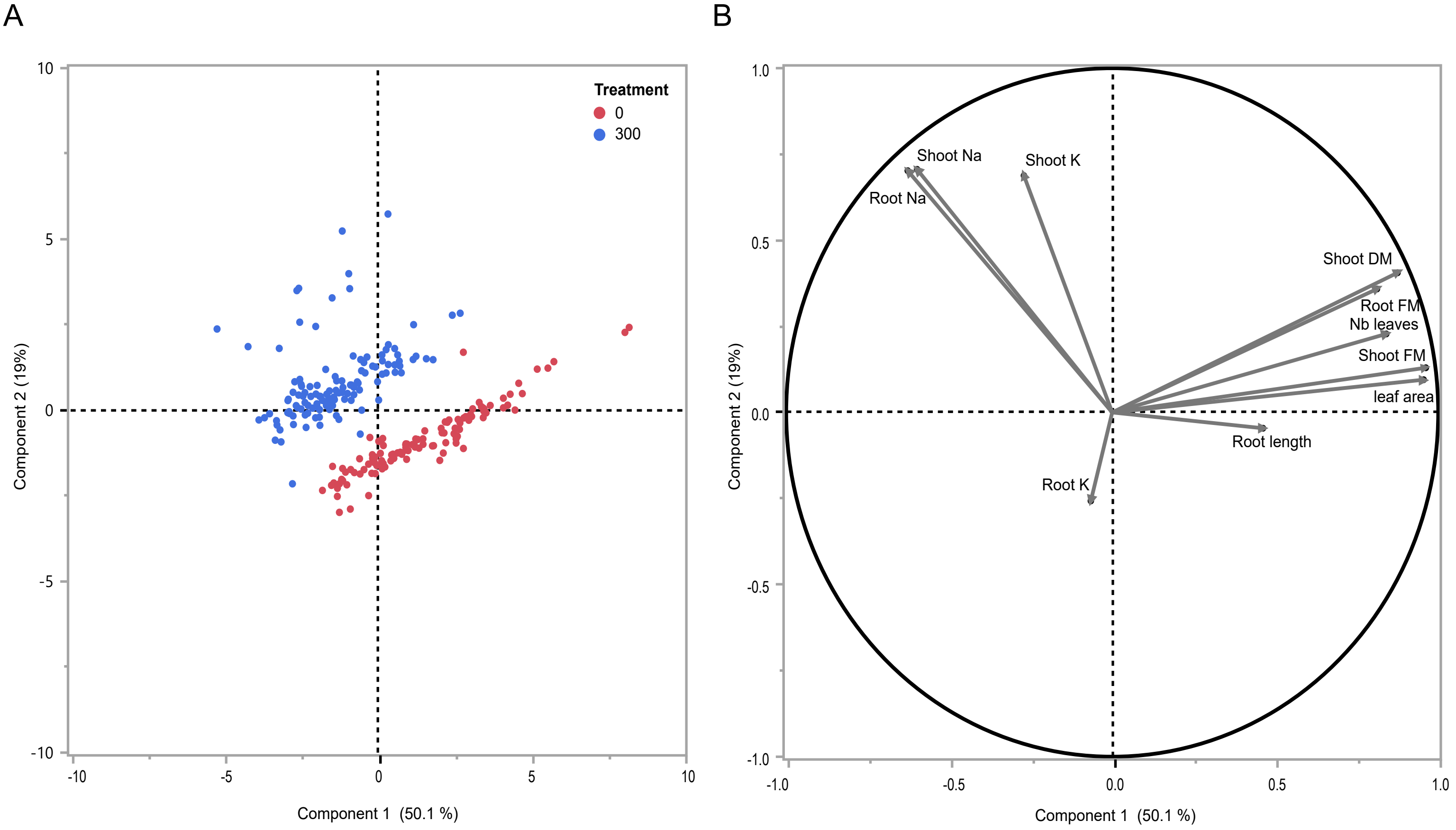

Supplementary Figure S2: Principal component analysis for traits hypothesized to contribute to salinity tolerance. (A) Scatter plot of PCA analysis with control (red) and salt treated (blue) *Chenopodium* accessions. Plotted points represent the average value for each measured trait for each accession. (B) Arrows indicate PCA analysis for traits indicated. All statistical analyses were performed in JMP.
