# Supplementary material for: Identification of Putative Transmembrane Proteins Involved in Salinity Tolerance in Chenopodium quinoa by Integrating Physiological Data, RNAseq, and SNP Analyses
Source: Front Plant Sci. 2017 Jun 21;8:1023. doi: 10.3389/fpls.2017.01023 (PMC5478719; doi:10.3389/fpls.2017.01023)
Supplement: Supplementary file 5 [file Image3.pdf]

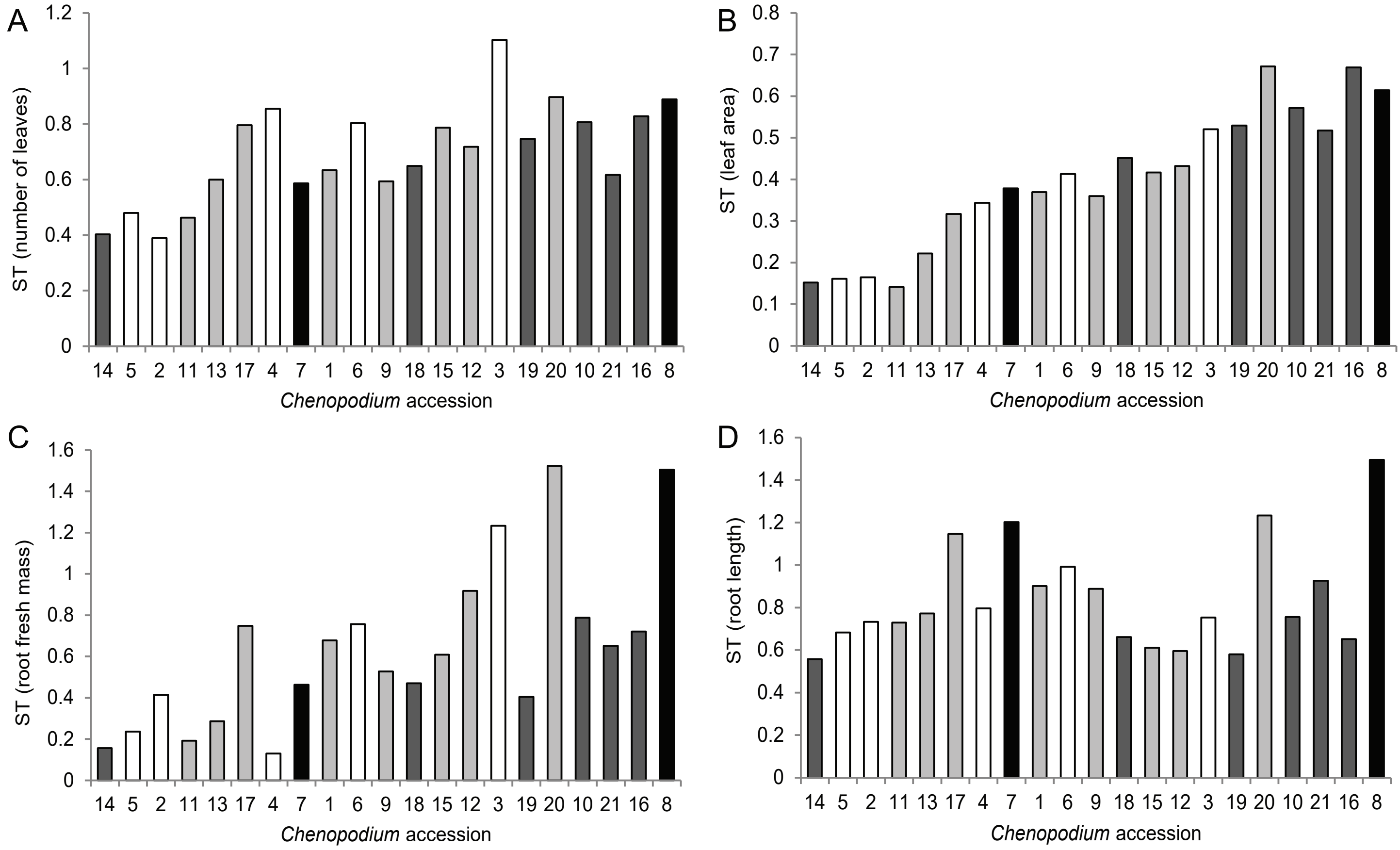

Supplementary Figure S3: Comparison of salt tolerance (ST) of different *Chenopodium* accessions. ST was calculated for (A) number of leaves, (B) leaf area, (C) fresh root mass and (D) root length. ST was calculated as the ratio of the trait during the salinity treatment compared to the control treatment. Accessions are described in Table 1. In short: (1) *C. q*. 0654; (2) *C. b*. PI 568156; (3) *C. b.* BYU 937; (4) *C. b.* PI 666279; (5) *C. b.* Ames 33013; (6) *C. b.* BYU 1314; (7) *C. h.* BYU 1101; (8) *C. h.* BYU 566; (9) *C. q.* CICA-17; (10) *C. q.* G-205-95DK; (11) *C. q.* Ollague; (12) *C. q.* Pasankalla; (13) *C. q.* Real; (14) *C. q.* Regalona; (15) *C. q.* Salcedo INIA; (16) *C. q.* Cherry Vanilla; (17) *C. q.* Ku-2; (18) *C. q.* Chucapaca (19) *C. q.* PI 634921; (20) *C. q.* Kurmi; (21) *C. q.* PI 614868.
