# Supplementary material for: Identification of Putative Transmembrane Proteins Involved in Salinity Tolerance in Chenopodium quinoa by Integrating Physiological Data, RNAseq, and SNP Analyses
Source: Front Plant Sci. 2017 Jun 21;8:1023. doi: 10.3389/fpls.2017.01023 (PMC5478719; doi:10.3389/fpls.2017.01023)
Supplement: Supplementary file 6 [file Image4.pdf]

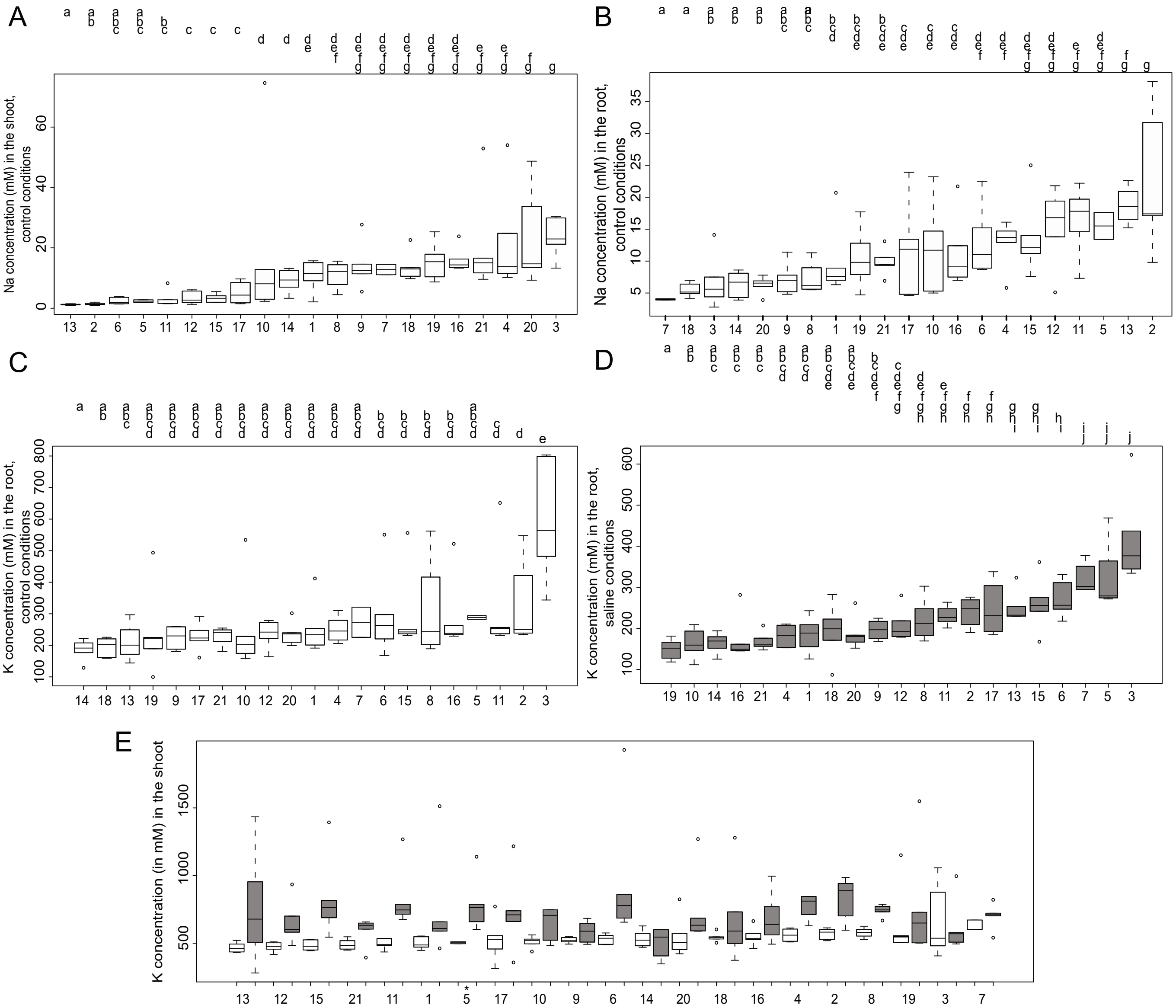

Supplementary Figure S4: Na and K distribution in salt stressed *Chenopodium* accessions. (A) Na concentration in the shoot and (B) root of control treated *Chenopodium* accessions. (C) K concentration in the root of control and (D) salt treated *Chenopodium* accessions. (E) K concentration in the shoot of control (in light grey) and salt (in dark grey) treated *Chenopodium* accessions; n=6, statistical analyses were performed as described in the methods section. F-statistic for (A), (B), (C) and (D) accession <0.0001, and for (E) accession 0.911 and treatment 0.062; levels not connected by the same letter are significantly different, $P \leq 0.05$. (Different layouts were chosen as the result of the fitted model suggesting in (E) no significant differences between accessions and treatment.)
